# Digital interactive experience- and game-based fall interventions for community-dwelling healthy older adults: a cross-disciplinary systematic review

**DOI:** 10.3389/fpubh.2024.1489258

**Published:** 2025-01-23

**Authors:** Celina Ciemer, Lisa Kröper, Thomas Jürgen Klotzbier, Sabiha Ghellal, Nadja Schott

**Affiliations:** ^1^Institute for Sport and Movement Science, University of Stuttgart, Stuttgart, Germany; ^2^Institute for Games, Stuttgart Media University, Stuttgart, Germany

**Keywords:** cross-disciplinary, interdisciplinary, fall prevention, exergame, user experience, game design, extended reality, interactive

## Abstract

**Introduction:**

Falls pose significant health risks to older adults, impacting their quality of life. Preventive strategies are crucial, as research shows that fall prevention interventions can effectively reduce fall risks. However, these interventions often suffer from low adherence and uptake. Digital, interactive interventions, incorporating experience-, and game-related aspects, offer a promising solution, making this topic inherently cross-disciplinary.

**Objective:**

This review aims to assess the current landscape of digital interactive experience and game-based fall interventions for community-dwelling, healthy older adults. It focuses on integrating Human Movement Science and User Experience & Game Design perspectives, emphasizing the cross-disciplinary nature of this research.

**Methods:**

We employed a cross-disciplinary literature search framework, searching the databases ACM-DL, IEEE-Xplore, ScienceDirect, PubMed, Scopus, and Web of Science. The review focused on healthy community-dwelling older adults (50+), including those at risk of falling. Excluded were studies involving chronic diseases, non-age-related impairments, other age groups, or individuals receiving care. Only digital, interactive fall prevention interventions without commercial software were considered. Studies published between 2000–2024 were included. A qualitative thematic synthesis was conducted, focusing on four categories: Objectives (O), Design and Development (D), Types of Intervention (T), and Evaluation Methods (E).

**Results:**

The search yielded 2,747 results, with 59 articles included in the final synthesis. Objectives were mainly driven by a combination of HMS and UXG rather than a single aspect. In Design and Development it was observed that concept-based design was scarce, with most being procedure-based. Descriptions of interventions frequently lacked specificity, particularly in-depth experience-related terminology and exercise descriptions. Evaluation methods were found to be more frequently informed by both HMS and UXG, although only four studies used a mixed-method approach to explore their interplay. Among included articles, most aspects incorporated both HMS and UXG across all four categories: O(*n* = 37), D(*n* = 37), T(*n* = 54), and E(*n* = 21).

**Conclusion:**

The review underscores the importance of digital interactive experience- and game-based fall prevention interventions. It highlights the need for enhanced cross-disciplinary collaboration between HMS and UXG to address gaps, such as the lack of a shared thesaurus and standardized guidelines, which are vital for improving transparency, reproducibility, and the refinement of these interventions.

## 1 Introduction

The global population is aging at an accelerating rate. As the World Health Organization (WHO) estimates by 2030, one in six people worldwide will be aged 60 or older, amounting to 1.4 billion. By 2050, this total is expected to increase to 2.1 billion (1).

Many health issues often accompany the aging process, collectively termed the *geriatric giants*. These problems significantly impact the quality of life of many older adults, including immobility, instability, cognitive impairment, and falls (2). Falls are common among older adults, with approximately one in three older adults experiencing a fall each year (3–6). The likelihood of falls is a considerable risk associated with aging, which tends to increase as motor and cognitive functions decline or when cognitive-motor disturbances intensify (7–10). The consequences of falls extend beyond physical injuries, as they can lead to a decline in self-confidence and self-esteem. This results in reduced activity and social isolation, affecting general well-being and quality of life but also accelerates physical decline and further exacerbates the aging process (3, 11, 12).

Therefore, it is of the utmost importance to implement early prevention interventions to reduce the risk of falls (11), making it a critical area of focus within Human Movement Science (HMS) research. The WHO emphasizes the promotion of healthy aging and proactive management of health risks as key strategies to assist older adults in maintaining their independence and active engagement in community life (13). A review of the literature on fall prevention reveals a significant body of evidence supporting the efficacy of various interventions. Meta-analyses have demonstrated that balance training can reduce fall risk by approximately 20% (14), while voluntary and reactive stepping has been shown to minimize the risk by up to 50% (15). Dual-task training has also been shown to reduce fall risk by a percentage that varies depending on the specific task (16).

Traditional fall prevention training has demonstrated shortcomings, particularly in poor adherence and motivation to conventional exercise interventions (17–19). The lack of positive experiences during interventions further contributes to this challenge (20). Digital interactive applications have emerged as enablers, offering innovative solutions for enhanced measurement tools and personalized interventions. By incorporating knowledge of game design and experience design (UXG), these applications aim to address the shortcomings of traditional human movement training by enhancing aspects such as adherence, engagement, usability, enjoyment, and providing meaningful experiences that extend beyond mere usage (21–29). Due to the variety of strategies and terminologies, we use the term *digital interactive experience- and game-based interventions* to encompass all types of applications.

Fall prevention through interactive experience- and game-based interventions is of great importance and has seen a notable increase in research activity over the last two decades. A scoping review from 2012 highlighted the growing interest in this area at the time (30). Since then, numerous systematic reviews have been conducted to evaluate the effectiveness of these interventions, which have shown positive impacts on outcomes. Notably, many of these studies have focused on randomized controlled trials (RCTs) or studies with control groups demonstrating significant improvements in balance, mobility, cognitive skills, and fall risk reduction among older adults (31–40). Other reviews have concentrated on specific areas, such as the technological aspects (41, 42), the design efforts in human-computer interaction (43), the movements and motor skills trained in exergaming interventions within the field of human movement science (44), the categorization of high-level physical activities (28), and the exploration of how motivation influences the effectiveness of interventions (45).

However, these existing studies often adopt an outcome-based approach limited to a single discipline. This results in a lack of comprehensive, cross-disciplinary evidence that is needed to fully capture the complexity of digital interactive experience and game-based fall interventions. Cross-disciplinary collaboration can be organized into different forms, varying in the processes used. The most commonly recognized forms are multidisciplinary, interdisciplinary, and transdisciplinary collaboration (46–51). However, so far there exists no generally accepted definition for each of these forms (52). The inherent nature of digital interactive experience- and game-based fall interventions allows them to transcend the research of individual disciplines. This nature makes cross-disciplinary collaboration essential for their development. In our analysis, we use the term cross-disciplinary as an inclusive concept that integrates all forms of collaboration.

It is of the utmost importance to understand these interventions comprehensively, encompassing their design, specific objectives, and the rationale behind their outcomes. This understanding is essential to ensure the reproducibility of these interventions and inform future refinements. Despite the importance of these aspects, a conspicuous deficiency exists in the extant literature, as to the best of our knowledge no systematic review has conducted a comprehensive examination of interventions from a cross-disciplinary perspective. Given the inherently cross-disciplinary nature of this topic, there is a pressing need for a systematic review that takes a cross-disciplinary approach from the outset, including a cross-disciplinary literature search to identify relevant articles.

In this systematic review, we investigate the current state of research on the cross-disciplinary topic of digital interactive experience- and game-based fall interventions in community-dwelling healthy older adults from the perspective of Human Movement Science and Experience & Game Design. In particular, we examine how the four key categories–objectives, design and development, types of digital interactive interventions, and evaluation methods–are addressed in the existing literature. We comprehensively overview these key categories and their application in current articles.

## 2 Related work

The Related Work section establishes the foundation for the thematic categorization presented in the results, drawing on relevant literature and studies. It provides a detailed explanation and definition of digital interactive experience- and game-based fall intervention.

### 2.1 Fall prevention interventions

The Prevention of Falls Network Europe defines a fall as an unintentional and sudden descent to the ground or a lower level due to an unintentional change in posture (53). Various studies have identified deficient motor and cognitive skills as contributing factors to falls, as evidenced by multiple studies (7). Fall prevention should address this deficiency by targeting various skills that can be trained differently. Central to these interventions are motor skills, particularly those related to standing, mobility, and gait. Standing primarily involves static postural control, which is the ability to maintain stability on a stable surface or when not engaged in locomotor activities, such as standing still while reading a book. Mobility and gait primarily involve dynamic postural control, which entails maintaining stability during movement or when the surface is unstable. Walking on uneven or unpredictable surfaces like gravel or grass can exemplify this. Research consistently shows that static and dynamic postural control are relatively independent motor abilities, each requiring targeted approaches to fall prevention (54). Although they are related, the mechanisms and skills required may differ, necessitating the implementation of distinct strategies for each type of postural control. Additionally, secondary mechanisms play a crucial role in these interventions. These include physiological parameters, particularly lower limb strength, and cognitive abilities such as complex attention, executive functions, and perceptual and locomotor functions. The nature of the task, whether it involves a single- or a dual-task, also serves as an important secondary mechanism influencing the effectiveness of these interventions. Dual-task situations, where an individual must perform two or more tasks simultaneously (e.g., walking while talking), often present a greater challenge to the cognitive and motor systems than single-task situations. This interference can lead to a reduction in performance, known as the dual-task cost, when a person has to perform two tasks simultaneously compared to performing each task separately (10, 55, 56). Therefore, they must be carefully considered in intervention design.

### 2.2 Digital interactive technologies

Digital interactive technologies are key enablers in modern applications as they expand the vast space of possibilities (57). Such technologies not only enable more precise measurement, but also allow for the development of solutions that can be tailored to suit the specific requirements and needs of individual users (58, 59). To navigate the diverse landscape of these technologies, we classify them based on two dimensions: the type of interaction and the degree of immersion they can provide. The type of interaction refers to how users engage with the technology. At its simplest, interaction occurs via touch-based mechanisms, such as tapping or swiping on a screen. These systems do not incorporate sensors for tracking body movements or recognizing gestures. Immersion, on the other hand, refers to the objective extent to which a system can involve users in an experience (60, 61) by technologies that modify reality by integrating digital elements into the physical or real-world environment (62).

Applications with very low immersion include touch-based interaction systems and screen-based virtual reality (VR). Touch-based interaction is used in applications which operate without additional sensors, relying solely on touch input via devices like tablets or smartphones with no other input methods available (e.g., mobile apps, training videos). Screen-based VR introduces sensor-driven interactions visualized on screens, such as world-fixed display (63) (e.g., kinect-based systems with screen). Moving up the spectrum, low immersion is typified by augmented reality (AR). AR overlays digital elements in the physical world and relies on interaction through a screen, such as AR glasses with screen-based interaction. Moderate immersion is achieved in mixed reality (MR), by merging real and virtual environments to offer a more immersive experience. Examples include systems that integrate physical devices like a force plate, ergometer, or Wii Balance Board with a screen. At the highest level of immersion, fully immersive VR provides the most immersive experience by fully enveloping users in a virtual environment and disconnecting them from the physical world. These systems typically employ head-mounted displays in combination with wearable sensors or body trackers.

### 2.3 Game design and user experience design

In the field of game design, crafting the player's experience is of paramount importance (64). While this experience is not the direct result of the game design, it is shaped by the player's unique choices and interactions during gameplay. Thus, the direct selection of game design elements and aspects according to the specific context and purpose plays a central role in creating positive and enjoyable player experiences (64–66). Player experiences are multifaceted, involving aspects such as the fulfillment of needs, attaining specific psychological states, and consequences of player actions (67, 68). The mechanisms triggered by these experiences may result in behavioral changes, motivation, or adherence.

In the context of games and the integration of health-related exercises and activities, there is an inconsistency in the terminology used by researchers (28). This inconsistency reflects uncertainty and disparate definitions, particularly between health-related and non-health-related domains (69). In this context a wide range of terms has been employed, such as *game-based exercising, active play video games, active gaming, exergaming, exertion games, health games*, or *embodied interactive video games* (69). It should be noted that the terms used in game design are connected to different underlying approaches. These approaches each pursue specific goals and employ distinct design processes (68, 70, 71). For example, exergames focus on both exercise- and game-related aspects (72–74), while in gamification, the game design is tailored to the non-game context (71, 75).

User Experience (UX) is vital in game design as well as in interactive non-game contexts (76, 77). In addition to usability (78), UX design considers emotional and psychological factors. It encompasses the user's perceptions and responses before, during, and after interacting with a product, considering emotions, beliefs, preferences, and behaviors (79). A positive UX is closely linked to fulfilling psychological needs, contributing to the user's overall well-being (80, 81).

## 3 Materials and methods

This systematic review was informed by the updated PRISMA 2020 guideline (82) and the ENTREQ statement for thematic synthesis as reporting standards (83). From the outset, a cross-disciplinary review was conducted using the cross disciplinary systematic framework for the literature search (Cris) (84). Subsequently, a qualitative approach was employed, using thematic synthesis to facilitate a systematic data analysis. This entailed a process of coding, developing descriptive themes, and generating analytic themes based on Thomas and Harden (85). The protocol was registered with the International Prospective Register of Systematic Reviews (PROSPERO, CRD42022344707). We updated the title and clarified definitions.

A cross-disciplinary research approach allows for examining a given topic from various perspectives, each contributing to a more nuanced understanding of the subject matter. It is essential to integrate the insights gained from different research areas and ensure that the literature search is designed to capture relevant studies from all angles, including their combinations and collaborations, to gain an accurate understanding of the current state of knowledge in this field. Accordingly, the search process itself must be cross-disciplinary. The framework proposed by Ciemer et al. (84) addresses this necessity by adhering to best-practice guidelines and reporting standards while integrating a preparatory process. The framework is designed to include research conducted across the full spectrum of cross disciplinary collaboration, including multidisciplinary, interdisciplinary, and transdisciplinary approaches. For scope determination, this framework developed PDICOS (Population, Design Approach, Intervention, Comparison, Outcomes, and Study Design) as a derivative of PICO (86, 87). Although PICO proved to be an excellent fit for HMS, where the emphasis is on evaluation and outcomes of interventions, it had to be adapted for UXG, where the focus is on the process and design (88).

### 3.1 Cross-disciplinary scope and eligibility criteria

The scope is defined by interpreting the research questions within each discipline. These interpretations are then combined into a unified cross-disciplinary framework. Accordingly, we employed PDICOS and derived the inclusion and exclusion criteria on this basis.

The following research questions informed our systematic research:

- **RQ1**: What types of objectives are addressed in digital interactive experience- and game-based interventions to prevent falls?
- **RQ2**: What types of digital interactive experience and game-based fall interventions are used?
- **RQ3**: What types of concepts and procedures are employed to develop and design interventions?
- **RQ4**: What type of evaluation methods and parameters were used to detect and measure:

(1) experience-related,
(2) cognition-related, and
(3) motor-related effects and outcomes?

This review included studies on community-dwelling, healthy older adults aged, including those prone to falls. Furthermore, we extended the age range to 50+ to be inclusive for fall prone people and different prevention strategies. We excluded participants with chronic diseases, non-age-related motor or cognitive impairments, other age groups, or individuals receiving care or injury rehabilitation. All design approaches were eligible. Interventions focused on fall prevention and training using digital interactive technologies including fully immersive VR, MR, AR, screen-based VR, and touch-based interaction. Studies using commercial products (e.g., Nintendo Wii or Xbox 360) were included only if no commercial software was used (e.g., the commercial product Wii Balance Board is allowed when the content is created within the research). Studies were excluded if they dealt with robotics, generic user research and technical implementation. Studies with or without control groups were eligible, emphasizing experience-, motor-, and cognition-related outputs. Only studies published in English or German from 2000–2024 were included, excluding systematic reviews, meta-analyses, books, dissertations, commentaries, gray literature, posters, and abstracts. Protocols were reviewed for supplemental data but not directly included.

### 3.2 Literature search: identification

#### 3.2.1 Creating the foundational search matrix

In the preliminary stage of the identification phase, the foundational search matrix (FSM) serves as the starting point for creating search strings (84). This is achieved by initially transforming PDICOS into categories, with each discipline defining explicit subcategories. Subsequently, each discipline assigns search terms to all subcategories, creating for every subcategory a specialized term depth. This FSM forms the cross-disciplinary thesaurus.

#### 3.2.2 Preparing the database search

We selected databases that consider each discipline's shared and unique objectives. In order to ensure a comprehensive representation of the cross-disciplinary topic, six major databases were selected, encompassing discipline-specific and cross-disciplinary resources. The databases included in the study were ACM DL, IEEE Xplore, ScienceDirect, PubMed, Scopus, and Web of Science. For each database its unique requirements for creating a search string were taken into account like query inputs, wildcards or specialized characters. It should be noted that ScienceDirect has a limit of eight operators. To align this with our search strategy, we used the most relevant terms from each discipline and terms related to our target audience. Based on these results, a custom filter was applied that covers the entire FSM. Furthermore, recommendations and guidelines for conducting searches, such as using controlled vocabulary or classification systems, were considered. These were integrated into the FSM for each database separately on an iterative basis. A golden bullet set was defined for each database, as recommended in Ciemer et al. (84) based on Zwakman et al. (89).

Using each FSM, database-specific search strings were constructed as boolean search queries. Ciemer et al. (84) outlined that the terms within each subcategory were connected with a logical OR, and each subcategory was combined with a logical AND. The search queries were validated by checking the inclusion of the golden bullet sets. All database-specific final search strings, such as Equation 1, which shows the logic for PubMed, and database-specific search matrices, are listed in the Supplementary material.


(1) $$\begin{array}{l} s=\left(\begin{array}{c} \left(\begin{array}{c} ”experience^{*}”\vee ”centered\;design”\vee ”interaction”\vee ”game^{*}”\\ \vee ”gami^{*}”\vee ”exergam^{*}”\vee ”user\;experience”\vee ”exer-game^{*}” \end{array}\right)\\ \wedge \\ \left(\begin{array}{c} ”fun”\vee ”emotion^{*}”\vee ”well-being”\vee ”wellbeing”\vee ”meaning^{*}”\\ \vee ”quality\;of\;life”\vee ”fear\;of\;falling”\vee ”usability”\vee ”engage^{*}”\vee \\ ”enjoy^{*}”\vee ”motivat^{*}”\vee ”risk\;of\;falling”\vee ”psychologic^{*}” \end{array}\right)\\ \wedge \\ \left(\begin{array}{c} ”mixed\;realit^{*}”\vee ”virtual\;realit^{*}”\vee ”augmented\;realit^{*}”\vee ”interactive”\\ \vee ”app”\vee ”vr”\vee ”ar”\vee ”mr”\vee ”xr”\vee ”extended\;realit^{*}”\vee ”kinect^{*}”\\ \vee ”computer”\vee ”system”\vee ”technolog^{*}”\vee ”tool”\vee ”wii”\vee ”application” \end{array}\right)\\ \wedge \\ \left(\begin{array}{c} ”fall”\vee ”falling”\vee ”fall-prevention” \end{array}\right)\\ \wedge \\ \left(\begin{array}{c} ”balance”\vee ”instability”\vee ”motor^{*}”\\ \vee ”cogniti^{*}”\vee ”physical”\vee ”movement” \end{array}\right)\\ \wedge \\ \left(\begin{array}{c} ”intervention”\vee ”treatment”\vee ”prevention”\vee ”training”\\ \vee ”exercise^{*}”\vee ”task^{*}”\vee ”therapy”\vee ”activit^{*}” \end{array}\right)\\ \wedge \\ \left(”elderly”\vee ”older\;adults”\vee ”senior^{*}”\vee ”age”\vee ”aged”\right) \end{array}\right) \end{array}$$

#### 3.2.3 Performing the database search

**Figure 1:**
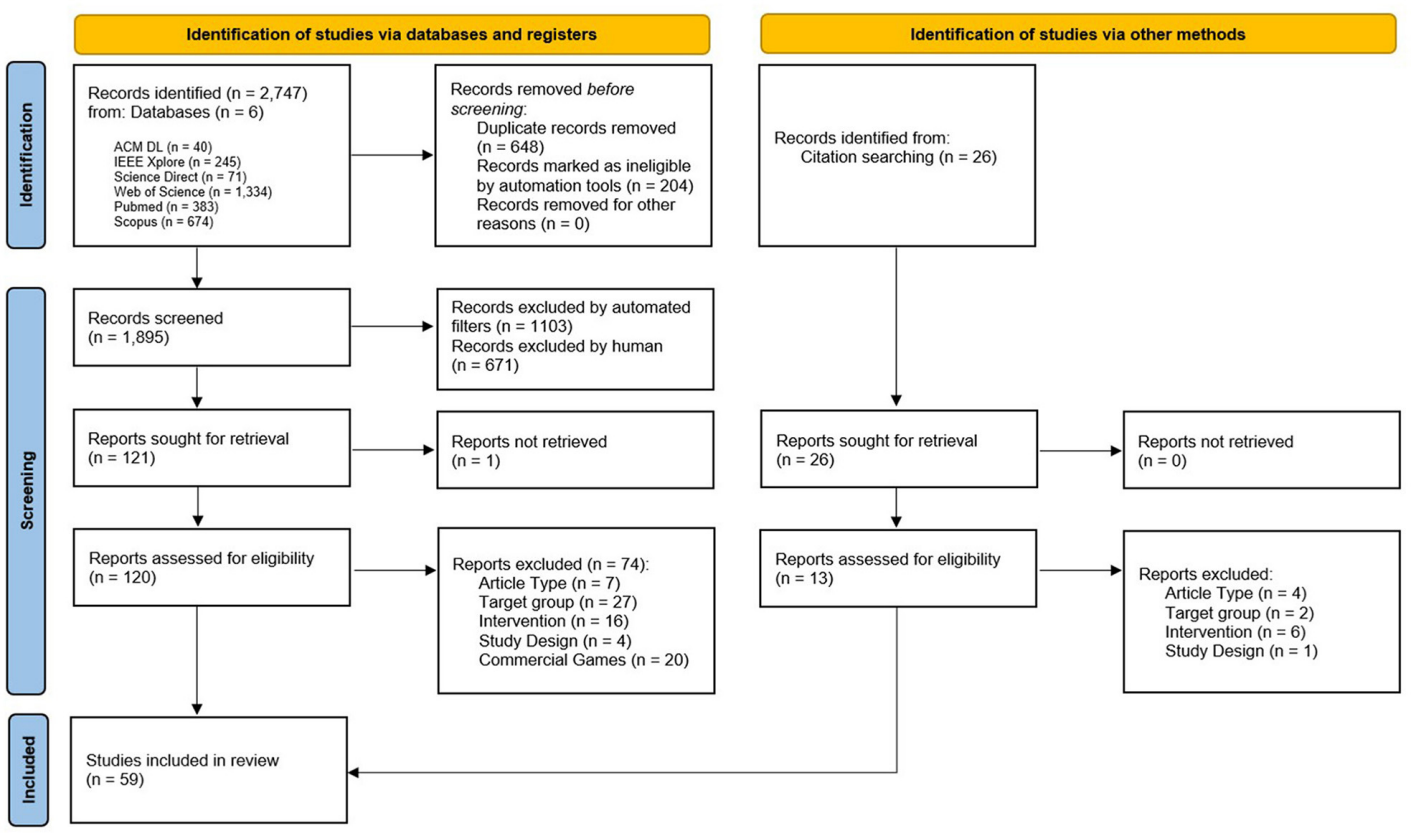
Flowchart of review process, according to the PRISMA guidelines (82).

The search was performed on January 2, 2024, and 59 articles were identified for inclusion (see Figure 1).

The search results were downloaded in BibTeX format, which was then used to extract relevant metadata. The metadata was then used to remove duplicates in a matching script, exclude literature types defined in the eligibility criteria, and remove all entries with the term 'review' in the title.

#### 3.2.4 Selection process and article screening

An automation tool was employed to apply additional filters. The filters were based on groups of terms that had been identified for exclusion (child terms, illness terms, and computer algorithms terms). A sample of *n* = 50 was randomly selected to review the title, abstract, and keywords to ensure the exclusion of irrelevant entries. Within the positive entries we also checked the metadata title, abstract and keywords in a table to identify further terms for exclusion. This process was repeated on an iterative basis. Two independent researchers from UXG and HMS (CC and LK) then screened the resulting entries, based on their titles and abstracts. The positive screened entries were then full-paper screened by the same researchers. In the event of a discrepancy regarding the inclusion or exclusion of a paper, a discussion was held, and, if necessary, an additional expert was consulted. Following the framework of (84) for cross-disciplinary searches, an article was included if one research field team deemed it to favor inclusion after discussion.

### 3.3 Data extraction and collection process

A customized form was developed for data extraction to capture relevant information. A metadata extraction was conducted using a script created in Jupyter Notebook, while the first author performed the qualitative data collection manually within the full texts.

Based on the research questions, an initial organizing categorization was developed to provide a structure for data extraction. This categorization was refined by incorporating the differentiation of aspects from UXG and HMS, where applicable. The resulting four key categories focus on the objectives, the design of the intervention to achieve these objectives, the interventions itself, and the intervention's evaluation.

An extraction scheme comprising predefined subcategories within a category was subjected to a pilot test in the initial iteration. The initial iteration was conducted along all included articles, given the considerable diversity in their focal points, specialized term depth, and thesauri. Accordingly, the extraction scheme was modified. Data was then collected according to the scheme. Any conflicts in classifications were resolved through iteration and joint discussion. In order to account for inconsistencies in reporting, along with our research questions, an article that did not contain information matching a subcategory was marked as *no entry* within this subcategory. Additionally, articles can have multiple entries in each subcategory.

The data extracted included subcategories from metadata and the four key categories, as shown in Table 1.

**Table 1:** Categories & metadata and subcategories used for data extraction.

**Categories & Metadata**	**Subcategories**
Metadata	year, authors, title, cross-disciplinary
Design and development objectives	objectives HMS, objectives UXG
Design and development of digital interactive interventions	concepts HMS, concepts UXG, procedures HMS, procedures UXG
Types of digital interactive interventions	types of fall prevention, type of task, types of exercises, types of experiences, types of technology
Evaluation methods of studies	evaluation methods HMS, evaluation methods UXG, shared evaluation methods, type of evaluation

### 3.4 Study quality assessment

In order to maintain focus on the specific objectives of this systematic review, a formal quality assessment using conventional tools has been deliberately avoided. This decision is based on the review's specific focus, which is not to assess methodological rigor or the effectiveness of interventions but to analyze and categorize articles from a cross-disciplinary perspective. The quality of the articles is evaluated according to the four key categories. These categories enable an evaluation of the conceptual strengths and relevance of the articles concerning the subject matter under examination.

### 3.5 Data synthesis

During the process of data synthesis, any data that was deemed to be excessively descriptive was subjected to coding. Themes were generated using both deductive and inductive approaches. Pre-existing definitions guided deductive approaches, whereas inductive approaches emerged directly from the data.

A comprehensive data cleansing process was undertaken to guarantee the consistency and precision of the data. This entailed the standardization of terminology to ensure uniformity across the dataset (e.g., harmonizing *think aloud* and *think-aloud* or *STS* and *Sit-To-Stand*). In the data synthesis process, unique items were extracted from the extracted data and codes. Furthermore, multiple entries within a subcategory were treated as unique items. When a topic was assigned to an item within a subcategory on more than one occasion, it was treated as a single entry. In the deductive case, the extracted elements were assigned to predefined themes. In the inductive case, descriptive and analytical themes emerged. These clustered themes were applied to the extracted data and codes, thus forming new subcategories. Two independent reviewers reviewed the resulting dataset manually to ensure accuracy and consistency.

Subsequently, to synthesize cross-disciplinary collaboration within and across various aspects, the data were analyzed using Jupyter Notebook for systematic counting and examination. The counting differentiates three distinct types within a given subcategory. (1) All entries style - each entry is counted individually, considering an article may have multiple entries, (2) Full entry style - the full entries of an article are counted as one entry, and (3) Top entry style - the differentiation is counted whether there is an entry or not. Furthermore, the data was analyzed by counting combinations of multiple subcategories, resulting in heatmaps, treemaps, time-dependent analysis, and network diagrams. In the case of heatmaps, percentages, and absolute numbers are expressed, whereas in the case of treemaps, only percentages are expressed. The time-dependent analysis was analyzed cumulatively or using a rolling mean of 5 to show trends. Network analysis examined the frequency of code combinations and the number of entries. All data is listed in the Supplementary material.

## 4 Results

### 4.1 Study characteristics

As a key characteristic in this cross-disciplinary review, we considered whether the studies explicitly identified themselves as cross-disciplinary, based on the terminology used by the authors to describe their work. Of the 59 included articles, 13.6% (*n* = 8) described themselves as interdisciplinary, multidisciplinary, or cross-disciplinary (72, 90–96). The majority, 86.4% (*n* = 51), did not use any term related to cross-disciplinary collaboration (97–147). The weighted mean age of the participants was 74.5 ± 7.9, calculated based on entries that included sample size, mean age, and standard deviation.

### 4.2 Results of synthesis

#### 4.2.1 Objectives of design and development

This category describes the types of objectives that guide the design and development process of digital interactive fall interventions **(RQ1)**. The key findings for HMS show that most studies focus on motor objectives. In UXG, one-third of studies lack any UXG objectives, while human related and digital training related objectives are evenly split. A trend toward combining HMS and UXG objectives is emerging.

##### 4.2.1.1 Human movement science objectives

In the context of HMS, four primary objectives for fall prevention have been identified: *motor, cognitive*, and *interference*. When treated as discrete entities, motor, or cognitive goals are assigned accordingly. The designation of interference is made when the objectives are described as dual tasks, when motor and cognitive tasks are to be performed simultaneously, or when motor-cognitive or cognitive-motor tasks are mentioned as objectives in the study. Full entry style counting is used for analysis.

**Figure 2:**
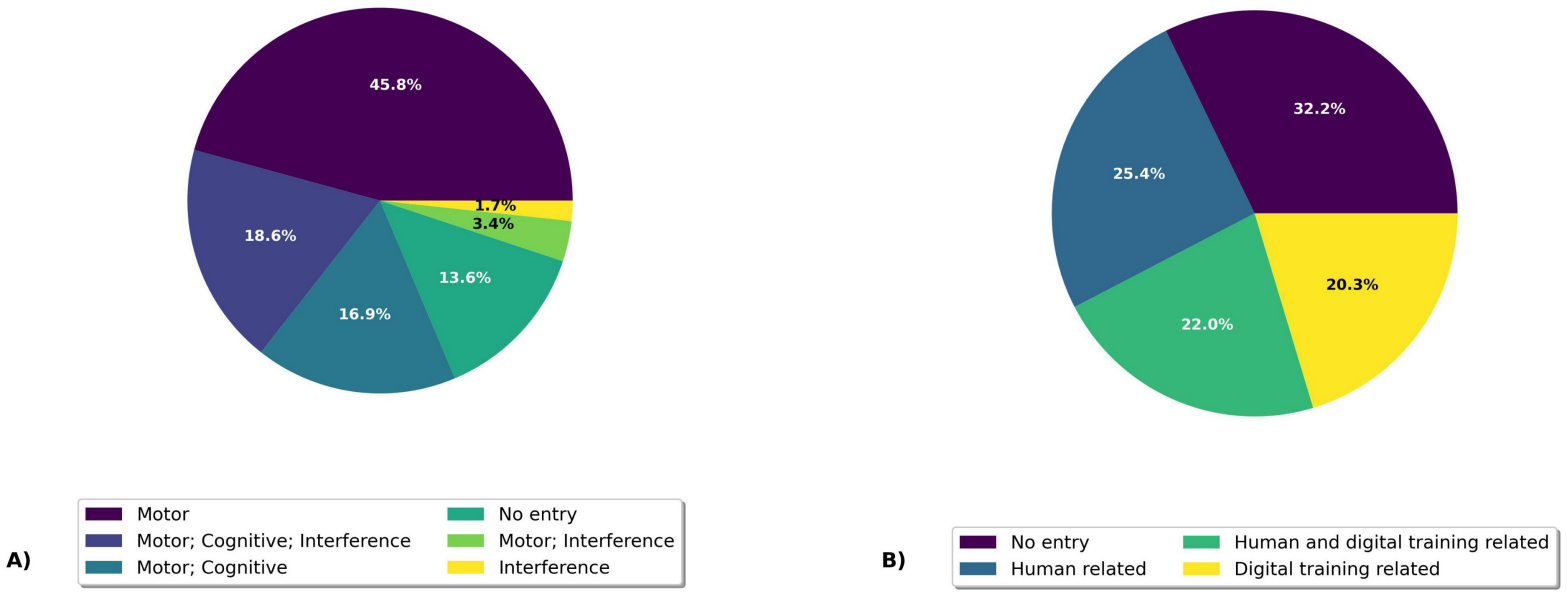
Percentage distribution of design and development objectives. **(A)** shows the human movement science objectives and **(B)** shows the user experience and game design objectives.

As shown in Figure 2A, 45.8% (*n* = 27) of the studies focused exclusively on motor objectives, while none focused solely on cognitive objectives. In 16.9% (*n* = 10) of the studies, both motor and cognitive objectives were addressed. Furthermore, 18.6% (*n* = 11) of the studies incorporated motor, cognitive, and interference objectives. Only a few studies addressed motor and interference goals (3.4%; *n* = 2) or solely interference objectives (1.7%; *n* = 1). 13.6% (*n* = 8) of the studies did not have HMS objectives.

##### 4.2.1.2 User experience and game design objectives

The subdivision of UXG objectives was deductively categorized, according to Retz et al. (68), into *human-related* and *training-related* objectives. The human-related objectives category concerns intended human-centered criteria and experiences, while the category of training-related objectives pertains to the perceptions of digital interactive training itself, including its properties, elements, and usability. Full entry style counting was used for analysis.

The analysis revealed that 25.4% (*n* = 15) of the articles focused exclusively on human-related objectives, while 20.3% (*n* = 12) concentrated solely on digital interactive training-related objectives. Additionally, 22.0% (*n* = 13) of the articles addressed both human-related and digital interactive training-related objectives. Notably, the largest proportion, 32.2% (*n* = 19), did not formulate any UXG objectives (see Figure 2B).

##### 4.2.1.3 Time-based analysis of objectives

**Figure 3:**
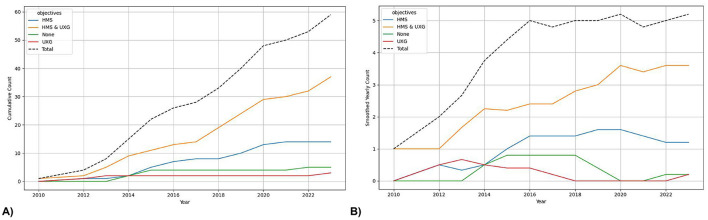
Time-based analysis of objectives. **(A)** shows the cumulative plot and **(B)** the trend with a rolling mean of 5.

The analysis of the Figures 3A, B reveals several important trends concerning the publication of studies with HMS and UXG objectives over time. Articles are labeled based on Figure 2: *HMS* if they include categories from Figure 2A, *UXG* for categories from Figure 2B, *HMS&UXG* if both appear, and *None* if neither is present. Since 2013, articles have consistently been published addressing HMS and UXG objectives, indicating a sustained interest in these combined objectives.

In contrast, the number of studies focusing exclusively on HMS objectives is lower than those combining HMS and UXG objectives. This number has been declining since 2020. While a few studies lacking clearly defined objectives were published between 2013 and 2015, these were rare occurrences, with an average of less than one study per year. Importantly, from 2015 to 2021, no studies were published without objectives. Finally, it should be noted that there have been very few studies with only UXG objectives. In fact, no studies with pure UXG focus were published between 2013 and 2022.

#### 4.2.2 Design and development of digital interactive interventions

The category *design and development of digital interactive interventions* examines the creation of these interventions **(RQ2)**. Two key themes have emerged from analyzing both the UXG and HMS aspects: *concept-based* and *procedure-based* intervention development. Concept-based intervention development emphasizes understanding and applying theories, models, frameworks, theoretical approaches, or ideas to inform decision-making processes and shape thinking. In contrast, procedure-based intervention development is centered on creating solutions to achieve specific outcomes or to address problems by following a series of defined steps, including design methods, research methods, and practical approaches. We used top entry style counting to ascertain the number of studies that employ a given concept or procedure, and and all entries style counting to analyze those groups further.

The summarized key findings are that design and development is predominantly procedure-based rather than concept-based. HMS primarily relies on concrete principles and strategies as well as research-based fall prevention. UXG emphasizes human-centered design procedures. A growing trend highlights increasing integration of HMS and UXG aspects.

##### 4.2.2.1 Design in human movement science

The subcategory *Design and Development HMS* was subjected to coding, and descriptive themes were derived from these codes. These were then divided into analytical themes.

At least one HMS concept for intervention development was used by 10.2% (*n* = 6) of the articles. The Framework for Design and Evaluation of Complex Interventions to Improve Health was used in two studies (118, 119). The remaining concepts were each employed once: Wickens' Theory of Shared Attentional Resources (127), Bottleneck Hypothesis (127), Dual Flow Model (129), Gentile's Taxonomy of Motor Skills (72), and FITT (Frequency, Intensity, Time, Type) Model (94).

**Figure 4:**
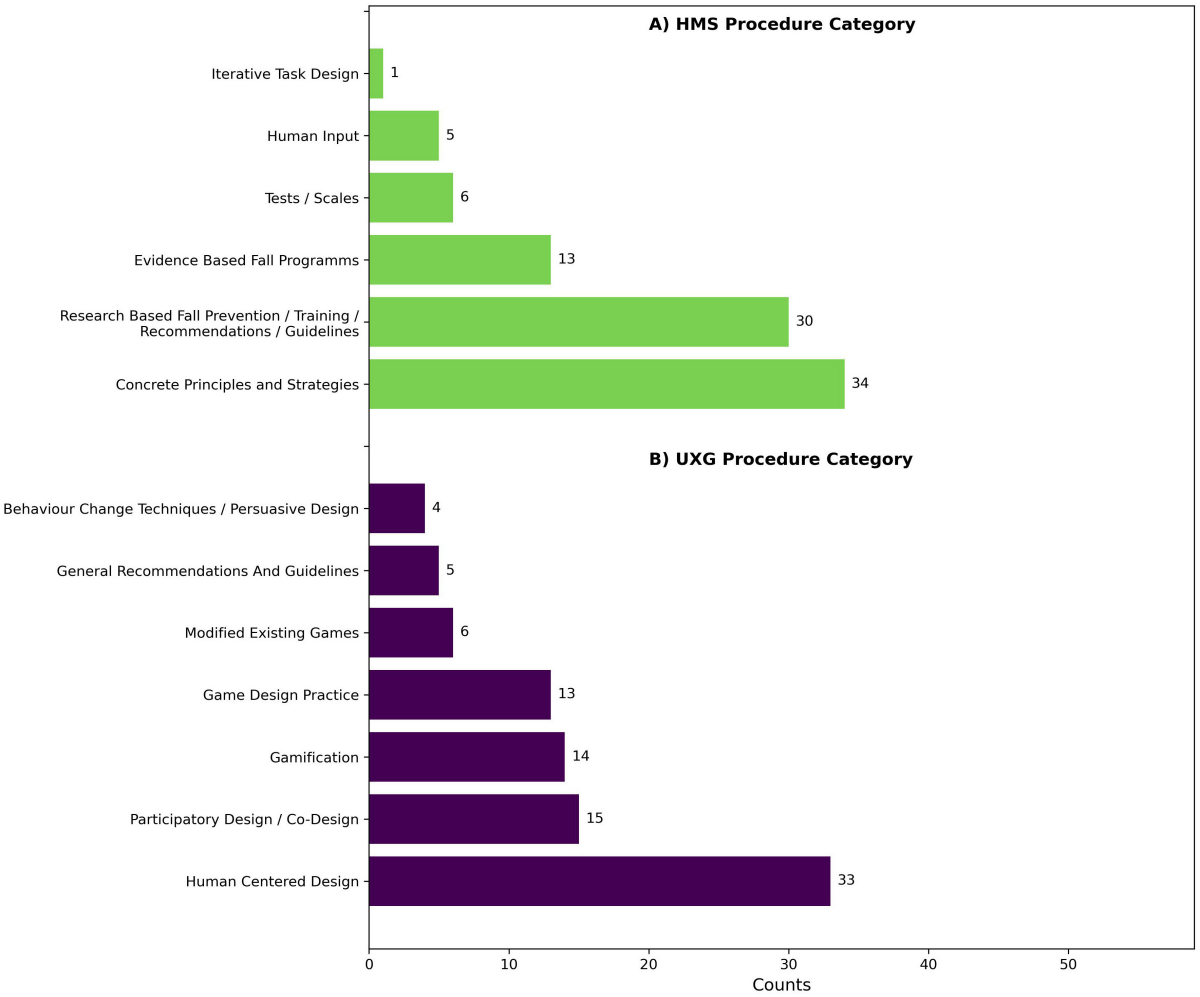
Barplots of descriptive themes from **(A)** human movement science procedures and **(B)** user experience and game design procedures in intervention development.

A minimum of one HMS procedure for intervention development was used by 86.4% (*n* = 51) of the articles. The codes were inductively divided into descriptive themes, with 89 entries related to procedures identified, indicating that some studies used more than one procedure. As shown in Figure 4A, 57.6% (*n* = 34) of the 59 studies were classified as *Concrete Principles and Strategies* for intervention design, which encompasses approaches such as a progressive increase in exercise difficulty, performance feedback, instructions, or point systems. Additionally, 50.8% (*n* = 30) were classified under *Research-Based Fall Prevention Training*, which involves the use of research findings and recommendations for fall prevention. Moreover, 22.0% (*n* = 13) were associated with *Evidence-Based Fall Programs*, including the Otago Exercise Program (OEP), Fitness and Mobility Exercise (FAME), and the Weight-Bearing Exercise for Better Balance (WEBB) program. An additional 10.2% (*n* = 6) used *Tests and Scales* such as the Timed Up and Go (TUG) or the Berg Balance Scale (BBS) as a foundation for the design. Additionally, 8.5% (*n* = 5) involved *Human Input*, such as interviews with healthcare professionals for HMS design. Finally, one procedure was related to *iterative task design* using Action Research.

When considering both HMS concepts and procedures, no study was found to be exclusively concept-based. A majority, 78.3% (*n* = 45), of the articles was purely procedure-based. Additionally, 10.2% (*n* = 6) of the studies used concepts and procedures as their foundation. 13.6% (*n* = 8) of the articles did not describe any HMS design foundation.

##### 4.2.2.2 Design in user experience and game design

The subcategory *Design and Development UXG* was coded, and from these codes, descriptive themes were derived and further divided into analytical themes.

15.3% (*n* = 9) of the articles used at least one UXG concept for intervention development. The codes of the UXG concepts were inductively assigned to descriptive themes. These themes contain a total of 13 entries. Of the 59 studies, 8.5% (*n* = 5) were related to *Human Needs and Embodiment*, such as the Hexad User Types, Self-Determination Theory, or Merleau-Ponty's concept of embodiment (72, 91, 106, 137, 139). Another 6.8% (*n* = 4) were categorized under *Behavior Change and Persuasion*, including for example the Behavior Change Technique Taxonomy v1 and Cugelman's persuasive gamification strategies (91, 96, 139, 142). Additionally, 3.4% (*n* = 2) were associated with *Flow Models*, including the Dual Flow Model and Game Flow Model (129, 135). Furthermore, one article focused on *Game Structure*, referencing models like the Mechanics Dynamics Aesthetics (MDA) Model, the Design Dynamics Experience (DDE) Model, and the Effectiveness, Enjoyment, Meaningfulness (EEM) Approach (72), and one article used gesture design theoretics (129).

74.6% ( *n* = 44) of the articles used at least one UXG procedure for intervention development. UXG procedure codes were inductively assigned to descriptive themes, with 90 entries categorized across the 59 studies, indicating that a study may use multiple procedures. Of the 59 studies (see Figure 4B), 55.9% (*n* = 33) were classified as *Human-Centered Design*, which included activities such as focus groups and interviews, references to human-centered design processes, or descriptions of specific process steps, such as understanding user needs, prototyping, testing, and reflection. A further 25.4% (*n* = 15) focused on *Participatory Design and Co-Design*, which included approaches such as participatory action research or participatory design workshops with older adults. Another 23.7% (*n* = 14) used *Gamification*, often identified by mentioning typical gamification elements such as rewards, point systems, badges, or scores. 14.4% (*n* = 13) were associated with *Game Design Practice*, including game themes, game rules, game goals, and storytelling. *Modified Existing Games* accounted for 10.2% (*n* = 6) of the studies, referring to modified versions of popular video games. *General Recommendations and Guidelines* were the foundation in 8.5% (*n* = 5) of the studies, while 6.8% (*n* = 4) used *Behavior Change Techniques and Persuasive Design*, such as scheduling exercises or providing motivational feedback.

When considering both UXG concepts and procedures, it was found that no study relied exclusively on the concepts. Instead, 59.3% (*n* = 35) of the studies were based solely on procedures, while 15.3% (*n* = 15) incorporated both procedures and concepts. Additionally, 25.4% (*n* = 9) did not specify any UXG design foundation.

##### 4.2.2.3 Interaction of design in human movement science with user experience and game design

The following analyses examine the interaction of design and development concepts and processes in the aspects of HMS and UXG.

**Figure 5:**
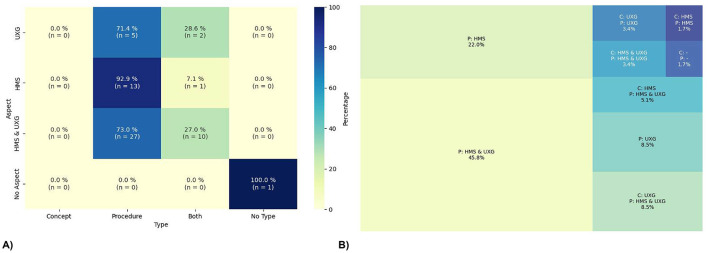
Interaction of design. **(A)** shows the heatmap of the interplay between aspects and types of the design and development, and **(B)** shows the treemap of the combination of aspects and types in the design and development. The concepts (C) as well as the procedures (P) are shortened in the map.

The heatmap (see Figure 5A) illustrates the distribution of entries across four design and development types-Concept, Procedure, Both (concept and procedure), and No Type-within the aspects UXG, HMS, UXG & HMS, and No Aspect. Notably, no articles are exclusively based on concepts. Concerning the aspect UXG & HMS, it can be observed that 73.0% (*n* = 27) of the articles are focused solely on Procedures, while 27.0% (*n* = 10) include both Concepts and Procedures. This aspect also contains the highest number of studies, with a total of 37 entries, indicating a high concentration of research in this combined aspect. A similar pattern is observed in the UXG aspect, with 71.4% (*n* = 5) of the articles dedicated to Procedures and 28.6% (*n* = 2) covering both Concept and Procedure. However, this category's total number of studies is notably lower, with only seven identified entries. In HMS, almost all articles, 92.9% (*n* = 13), focus exclusively on Procedures, with just one study (*n* = 1) including both Concepts and Procedures. Additionally, it is important to note that one study does not describe a type of design related to UXG or HMS.

The treemap (see Figure 5B) illustrates the integration of the UXG and HMS aspects with the types for Concepts (C) and Procedures (P). The percentages are calculated based on the total number of studies (*n* = 59). The results indicate that 45.8% (*n* = 27) of the studies used solely Procedures from both HMS & UXG. Additionally, 22.0% (*n* = 13) focused solely on Procedures but only from the HMS perspective, while 8.5% (*n* = 5) also used Procedures exclusively from the UXG aspect. It should be noted that no studies focused exclusively on Concepts were identified. However, 8.5% (*n* = 5) of the studies employed a UXG Concept with an HMS and UXG Procedure, while 5.1% (*n* = 3) used a HMS Concept combined with an HMS and UXG Procedure. Smaller groups of articles can be identified, comprising 3.4% (*n* = 2) of the total sample that employs both a UXG Concept and a UXG Procedure and 1.7% (*n* = 1) that used HMS Concepts and Procedures. One article does not use any HMS or UXG Concepts or Procedures. Furthermore, it is noteworthy that only two articles encompass the complete set of HMS and UXG Concepts and Procedures.

##### 4.2.2.4 Time-based analysis of design and development

**Figure 6:**
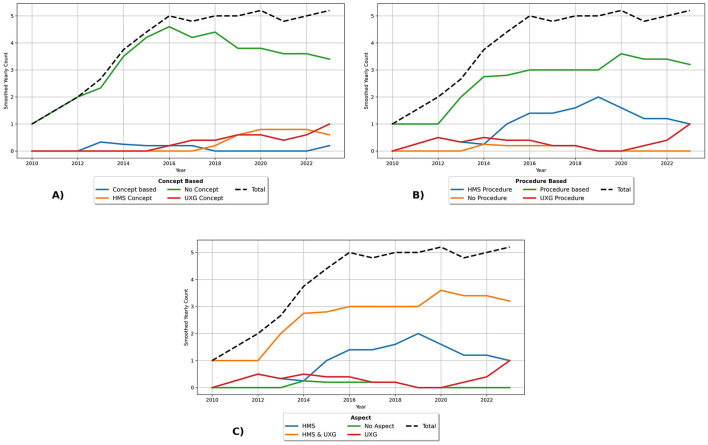
Time-based analysis of Design and Development with a rolling mean of 5. **(A)** shows the trend for concept-based aspects, **(B)** for procedure-based aspects and **(C)** for overall design aspects.

Figure 6A illustrates the trend of concept-based aspects over time, distinguishing between those originating from UXG, HMS, both, or neither. Until 2016, most articles were developed without incorporating concepts from either UXG or HMS. Subsequently, the trend has been steadily declining, and more articles with concepts have been published. Among the articles that do include concepts, there has been a notable increase in the use of UXG Concepts since 2015. From 2017 to 2020, HMS Concepts were also employed, but their usage has since plateaued. Concepts combining both aspects, UXG and HMS, have remained consistently low and are nearly non-existent over the observed time period.

Figure 6B illustrates the trend of procedure-based aspects over time, distinguishing whether the procedure originates from UXG, HMS, both or if no procedure is present. Prior to 2014, the majority of procedure-based articles were developed primarily from both HMS and UXG aspects. Following this period, the number of studies continued to increase but reached a plateau. From 2014 to 2019, there was an increase in the publication of articles focusing solely on HMS Procedures, after which the trend declined steadily. Since 2020, there has been an increase in studies relating exclusively to UXG Procedures. Notably, there are very few articles that do not incorporate any procedures.

Figure 6C illustrates the trend of the overall design aspects over time, distinguishing between those originating from UXG and HMS, as well as both or if no procedure is present. Until 2014, most articles were developed from UXG and HMS. Subsequently, the trend of studies increased further but reached a plateau. From 2014 to 2019, there was a notable increase in articles focusing exclusively on HMS. However, following this period, the trend exhibited a steady decline. Since 2020, there has been an increase in studies relating exclusively to UXG. Notably, the figure referenced above Figure 6C, which illustrates the development of the overall design aspects over time, resembles Figure 6B, which demonstrates the trend of procedure-based aspects over time. This is because there is no entry where Concepts have more aspects or at least another aspect than Procedures.

#### 4.2.3 Types of experience and game based digital interactive interventions

This category examines the various types of digital interactive experiences and game-based fall interventions and how these are described in the articles **(RQ3)**. Given the focus of our research, each intervention comprises a type of fall prevention, a type of intended experience, and a digital interactive technological enabler.

The key results in this category are that types of fall prevention do not focus on gait within dynamic postural control. The terms used to describe types of experiences are applied arbitrarily across studies. Regarding technologies, VR screen-based systems, very low-immersive technologies, are most commonly employed. However, a notable trend shows an increase in MR applications.

##### 4.2.3.1 Types of fall prevention

The types of fall prevention were differentiated according to the skills critical to reducing the risk of falling. Based on related work, the following descriptive themes were used: *stand, mobility, gait, physiological parameters*, and *cognition*. All entries style was used for counting.

**Figure 7:**
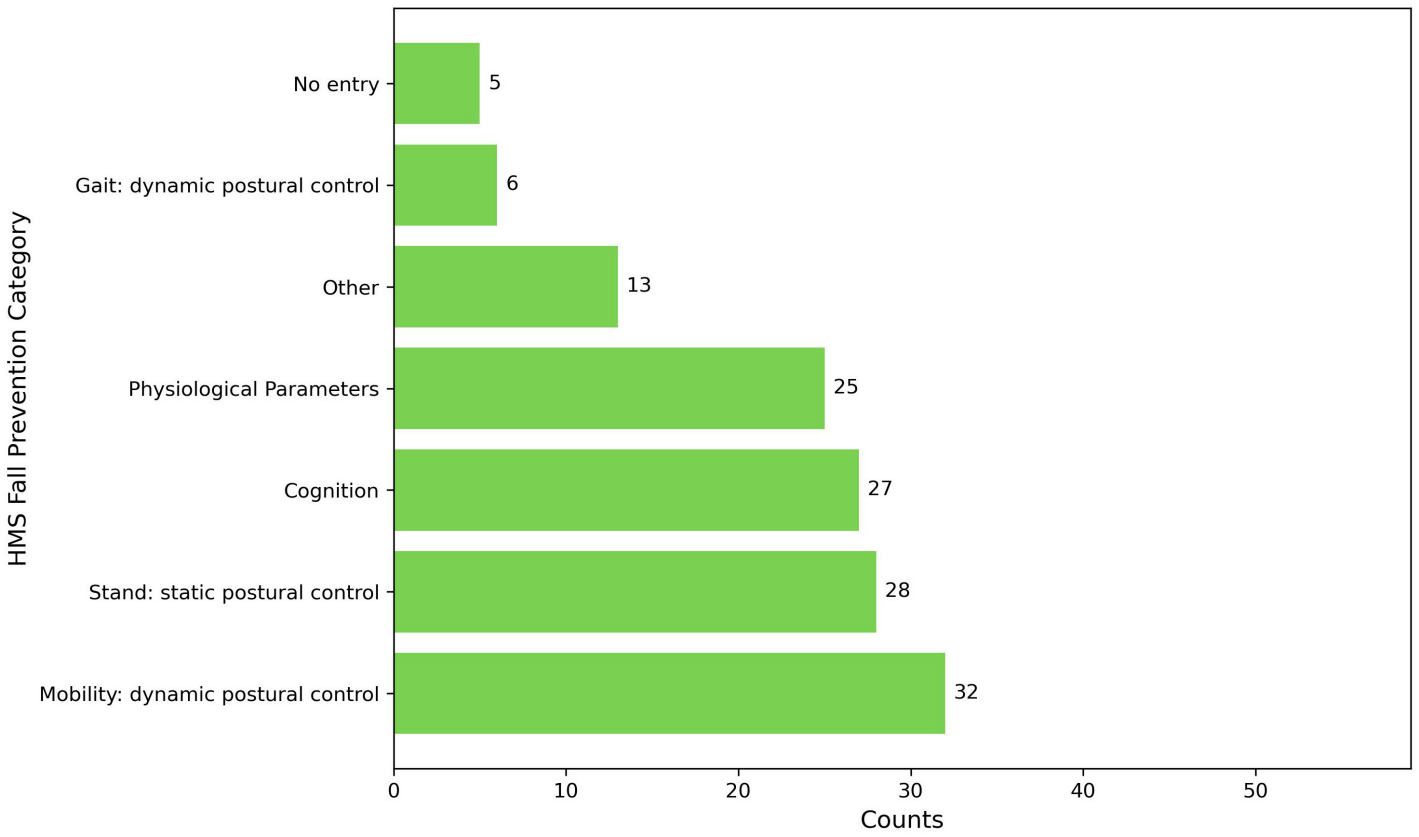
Barplot of types of fall prevention.

A total of 136 entries about intervention options for falls were identified across the 59 articles. As shown in Figure 7, mobility, which involves dynamic postural control, was addressed in 54.2% (*n* = 32) of the articles. This category encompassed a range of exercises, including stepping in various directions, side-stepping, dance movements, and standing on unstable surfaces. Another aspect of dynamic postural control, namely gait, was addressed in 10.2% (*n* = 6) of the articles. These articles referred explicitly to walking in different forms and under various conditions, including forward, backward, in circles, at different speeds, and when avoiding obstacles. Stand, which involves static postural control was addressed in 47.5% (*n* = 28) of the articles, with exercises designed to maintain stability in various positions (e.g., semi-tandem stance, or tandem stance), maintain balance while standing on one foot, and perform functional reaching and weight transfer exercises while standing. In addition, cognition was explored in 45.8% (*n* = 27) of the articles, primarily through the implementation of exercises designed to enhance complex attention, executive functions, and perceptual motor skills. Physiological parameters were discussed in 42.4% (*n* = 25) of the articles, focusing on exercises designed to improve lower body strength and muscle activity. A smaller portion, 22.0% (*n* = 13), fell into the Other category, encompassing various activities that did not fit into the other classifications like cycling or education. Lastly, 8.5% (*n* = 5) of the articles did not specify which skills were addressed, categorized as *No entry*.

In addition to these themes, another descriptive theme set targets the nature of a task. This analysis considered whether the interventions focus on single-tasks, dual-tasks, or a combination of both, using full entry style counting. It was found that 39.0% (*n* = 23) of the articles used single-tasks exclusively, 30.5% (*n* = 18) of the studies incorporated both single- and dual-tasks, and 18.6% (*n* = 11) focused exclusively on dual-tasks. In 11.9% (*n* = 7) of the studies, there was no description of the nature of task used.

The task descriptions were analyzed, which led to the inductive derivation of two analytical themes that applied to motor components addressed in most of the articles. The first theme centered on the classical description of HMS exercises and the level of detail provided, which was categorized into three codes: *Basic description (a simple outline of the task), General labeling (naming the task type)*, and *No description*. Full entry style counting was used for analysis. The second theme examined the explanation of the transfer in the application, which was categorized as either *explained* or *not explained* using top entry style counting.

For the descriptions of the classical motor exercises, 69.5% (*n* = 41) were categorized as basic descriptions, while 13.6% (*n* = 8) were categorized as general labeling. Notably, none of the articles reported how the exercises were integrated into a specific training plan within the application, and 16.9% (*n* = 10) lacked any description. Regarding the transfer of the exercises into the application, 25.4% (*n* = 15) included a description. It is important to note that these studies did not necessarily include a classical description.

##### 4.2.3.2 Types of experience and types of digital interactive technologies

**Figure 8:**
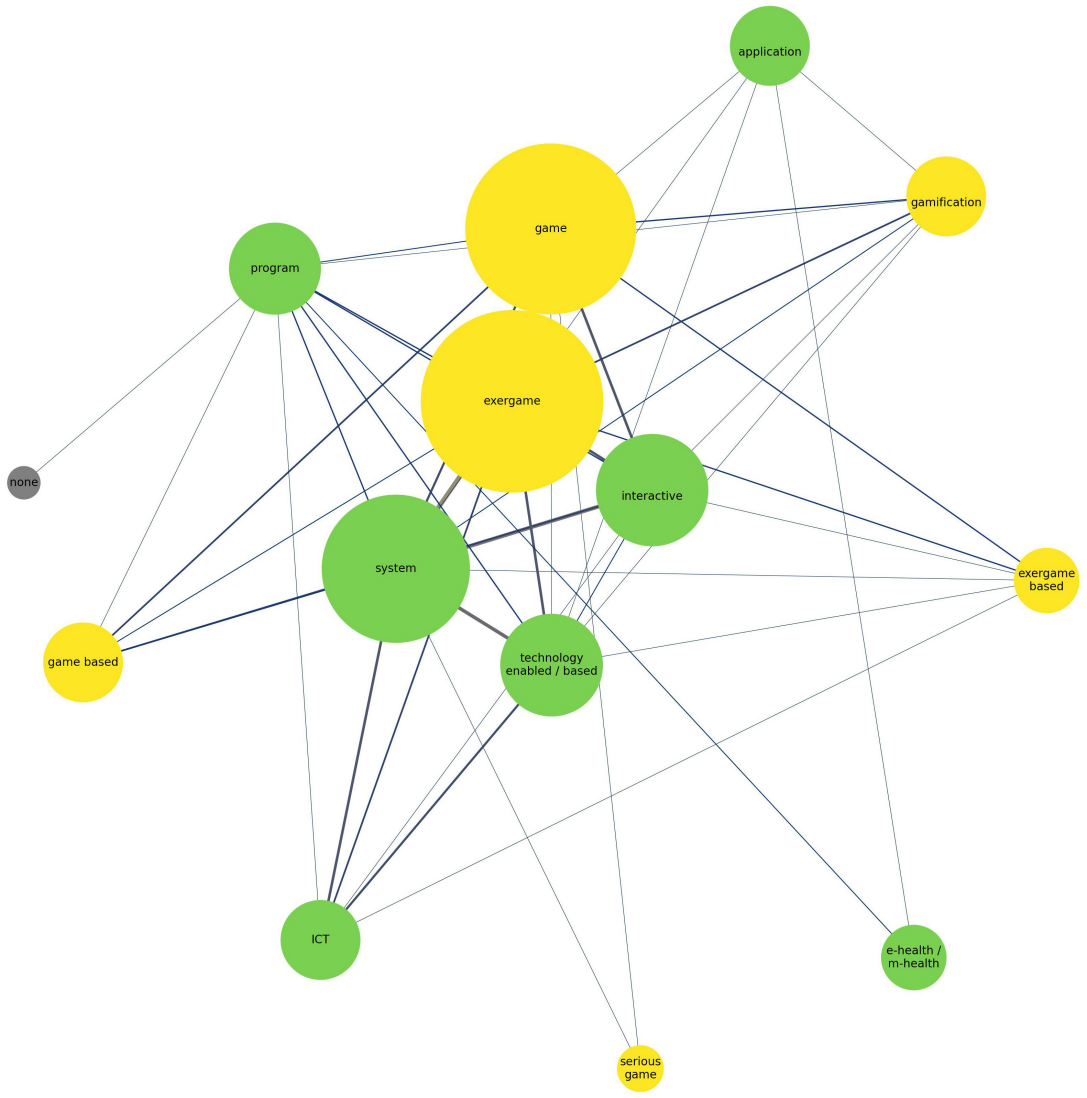
Network diagram of application naming. Yellow nodes represent game-related terms, green nodes non-game-related terms, and gray nodes unspecified terms. Node size reflects frequency, and line thickness indicates relationship strength.

The use of digital interventions is inherently tied to the experiences they provide for participants. In the context of this review, these experiences are coded according to whether the application is *game-related* or *not*. A distinction was determined by analyzing the names of the applications using top entry style counting. An application was classified as game-related if the article referenced any gaming terms. Notably, 79.7% (*n* = 47) of the studies involved game-related experiences. Further analysis consisted of deconstructing the names of the applications and coding them according to different types. It is important to note that a single application can receive multiple codes. Figure 8 presents a network diagram illustrating the frequency of code combinations and the amount of entries found in application names. The diagram uses colors to differentiate whether a code is game-related, not game-related, or does not contain a specific technology label. The size of each bubble indicates the frequency with which a term appeared in our analysis, while the shading and thickness of the lines connecting the bubbles indicate the strength of these relationships. Additionally, the spatial positioning of the bubbles reflects the strength of their relationships. The most frequently occurring term in the analysis is *exergame*, followed by *game*. Other game-related terms include *gamification, exergame-based, game-based*, and *serious game*. In contrast, non-game-related terms appeared less frequently, with *system* being the most common, followed by *interactive* and *technology (-enabled/-based)*, then *Information and Communication Technology/Platform (ICT), e-health/m-health, program*, and *application*. Exergame shows a very strong connection with game, a strong association with system, and moderate frequent connections with interactive, gamification, and technology (-enabled/-based). Moreover, exergame, game, and system exhibit numerous associations with other codes.

Technological enablers create a vast space of possibilities for applying design concepts and procedures and can be categorized based on their type of technology and interaction possibilities. Our analysis made it apparent that the terminology around these technologies is used ambiguously in the existing studies. To address this, we adopted the definitions from IxDF IDF (62), Milgram and Kishino (148), and Slater (60) as outlined in the related work section and applied a deductive clustering approach that distinguishes between the type of interaction and degrees of immersion resulting in the categories: touch-based interaction, screen-based VR, AR, MR and fully immersive VR. All entries style counting was used for analysis.

**Figure 9:**
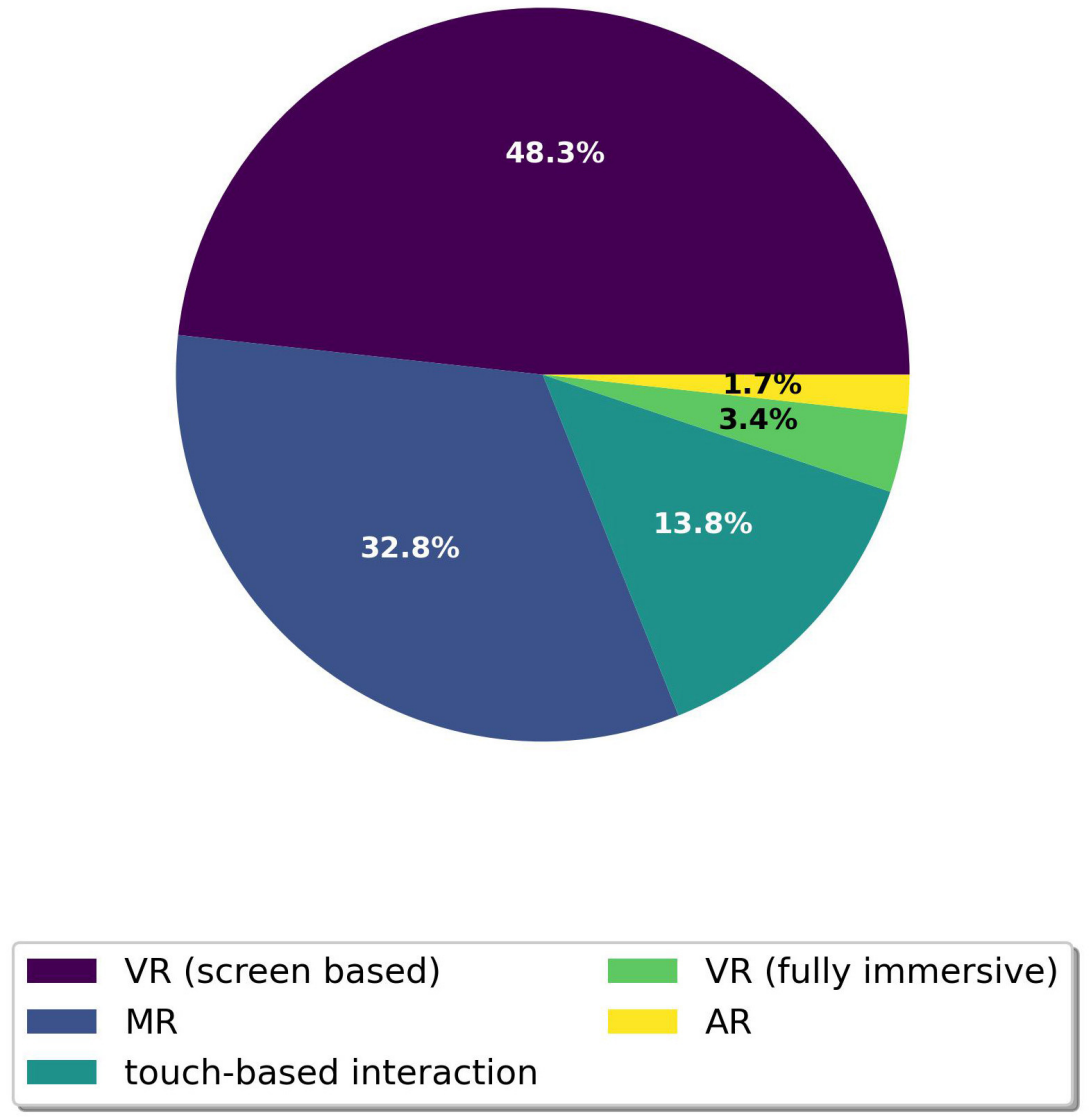
Pie diagram of technological enablers used.

Figure 9 shows that nearly half of the articles, 48.3% (*n* = 27), employed VR in a screen-based format, while 32.8% (*n* = 18) incorporated MR. Touch-based interactions were featured in 13.8% (*n* = 8) of the studies, and only 3.4% (*n* = 2) used fully immersive VR environments. AR was the focus of one article. Three articles do not describe a specific technological enabler but were included in this review because they represent direct process steps in developing digital interactive fall interventions.

##### 4.2.3.3 Time-based analysis of types of digital interactive technologies

**Figure 10:**
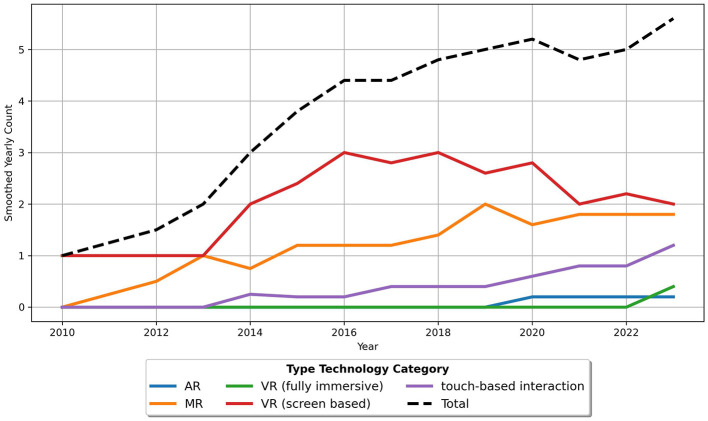
Trend with a rolling mean of 5 for technology usage in applications.

Figure 10 presents a trend diagram illustrating the technologies used in the applications. It is important to note that a single article may include more than one technology. The diagram reveals that screen-based VR showed a positive trend, with continuous growth until 2016, reaching up to three articles per year, but has since seen a global decline. MR demonstrates a consistently positive trend throughout the entire period, remaining slightly below the use of screen-based VR until 2023. Touch-based interaction has been steadily increasing since 2013, positioned below MR. Fully immersive VR is a more recent addition, only appearing in studies from 2023 onwards. AR has played a minor role and was mentioned for the first time in 2019.

#### 4.2.4 Evaluation methods of studies

The category *evaluation methods* refers to the assessment of interventions and the methods chosen within an article **(RQ4)**. During the analysis, it became evident that distinguishing between UXG and HMS was insufficient, as some methods can be applied to both aspects. For this reason, *Shared evaluation methods* were introduced to capture the methods that can be assigned to both areas. The entries were categorized inductively, with attention to distinguishing between qualitative and quantitative methods. We applied descriptive themes to the methods. To analyze the evaluation methods for HMS and UXG, we used top entry style counting to get the number of evaluation methods used and all entries style counting to analyze the used methods further. For further analysis, we created analytical themes for the types of evaluation methods and used full entry style counting.

The key results in this category are that HMS primarily relies on quantitative methods, while UXG uses a balanced mix of qualitative and quantitative methods. Shared methods are rarely employed. Over time, evaluation methods have predominantly been separated for HMS and UXG, but a recent trend shows increasing use of combined methods.

##### 4.2.4.1 Evaluation methods of human movement science

**Figure 11:**
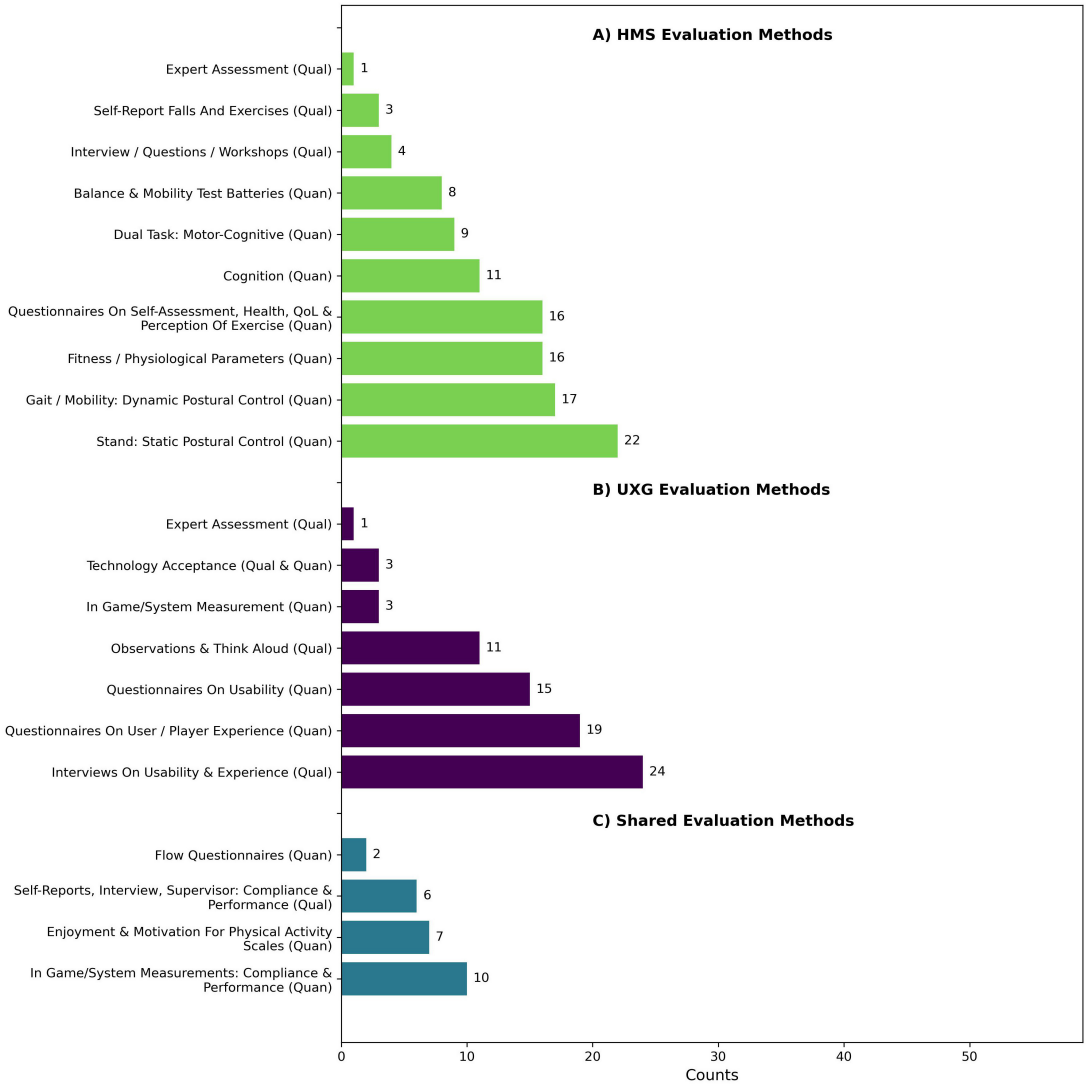
Counts of descriptive themes of evaluation methods of **(A)** human movement science, **(B)** user experience and game design and **(C)** shared evaluation methods.

Figure 11A shows that HMS evaluation methods were used in 57.6% (*n* = 34) of the articles, resulting in a total of 107 identified entries. Of the 59 articles, 37.3% (*n* = 22) focused on *Stand: Static Postural Control tasks (quan)*, using instruments such as the Berg Balance Scale (BBS), Functional Reach Test (FRT), Y-Balance Test, or Choice Stepping Reaction Time (CSRT). *Gait/Mobility: Dynamic Postural Control tasks (quan)* were assessed in 28.8% (*n* = 17) of the articles using methods such as the Timed Up and Go (TUG) test and gait kinematics. Another 27.1% (*n* = 16) looked at *Fitness/Physiological Parameters (quan)*, including tests such as the Sit to Stand (STS) test, the 30-Second Chair Stand (CS-30) test, and heart rate monitoring. In addition, 25.4% (*n* = 15) of the studies used *Questionnaires on Self-Assessment, Health, Quality of Life, and Perception of Exercises (quan)*, such as the Borg Scale of Perceived Exertion, the Iconographical Falls Efficacy Scale, and the International Physical Activity Questionnaire (IPAQ). *Cognition* was assessed in 18.6% (*n* = 11) of the articles using tools such as the Trail Making Test (TMT) and the Victoria Stroop Test (VST). Furthermore, 15.3% (*n* = 9) of the studies focused on *Dual-Task: Motor-Cognitive (quan)*, with instruments such as the TUG under dual-task conditions, the Stroop Stepping Test (SST), and counting backward while walking 10 meters. *Balance and Mobility Test Batteries (quan)*, such as the Short Physical Performance Battery (SPPB) and Physiological Profile Assessment (PPA), were used in 13.6% (*n* = 8) of the studies. *Interviews, Questions, and Workshops (qual)* were conducted in 6.8% (*n* = 4) of the studies to assess subjective improvement and physical and cognitive involvement. *Self-Report Falls and Exercises (qual)* were recorded in 5.1% (*n* = 3) of the studies, for example through fall diaries. Finally, one study included *Expert Assessment (qual)* on implementing HMS design approaches.

##### 4.2.4.2 Evaluation methods of user experience and game design

Figure 11B shows that UXG evaluation methods were used in 61.0% (*n* = 36) of the articles, resulting in a total of 76 identified entries. Of the 59 studies, 40.7% (*n* = 24) of the studies employed *Interviews on Usability & Experience (qual)*, in various forms such as semi-structured, structured, or discussions. Additionally, 32.2% (*n* = 19) used *Questionnaires on User / Player Experience (quan)*, including instruments such as the Play Experience Scale (PES), the Game Experience Questionnaire (GEQ), and the User Experience Questionnaire (UEQ). Furthermore, 25.4% (*n* = 15) used *Questionnaires on Usability (quan)*, such as the System Usability Scale (SUS) and custom usability questionnaires. *Observations & Think Aloud (qual)* during interaction with the application were used in 18.6% (*n* = 11) of the studies. *Technology Acceptance methods (qual + quan)* were employed in 5.1% (*n* = 3) of the studies, while another 5.1% (*n* = 3) used *In-Game/System Measurement (quan)*, such as game scores and point rates. Finally, one article used *Expert Assessment (qual)* on implementing UXG design approaches.

##### 4.2.4.3 Shared evaluation methods

As illustrated in Figure 11C, shows that in 30.5% (*n* = 18) of the articles, shared evaluation methods were used, leading to the identification of 25 distinct entries. Of the 59 studies, 16.9% (*n* = 10) involved *In-Game/System Measurements (quan)* and concentrated on matters of compliance and performance. Additionally, 11.9% (*n* = 7) used *Scales for Assessing Enjoyment, Motivation, and Well-being in Physical Activity (quan)*, such as the Physical Activity Enjoyment Scale (PACES) or the COMPAS-W scale of Wellbeing. Furthermore, 10.2% (*n* = 6) relied on *Self-Reports, Interviews, and Supervision* to evaluate compliance and performance using diaries, attendance protocols, counting, or structured in-person interviews. Finally, 3.4% (*n* = 2) employed *Flow Questionnaires*, including the Flow State Scale (FSS) and Activity Flow State Scale (AFSS).

##### 4.2.4.4 Types of evaluation methods

**Figure 12:**
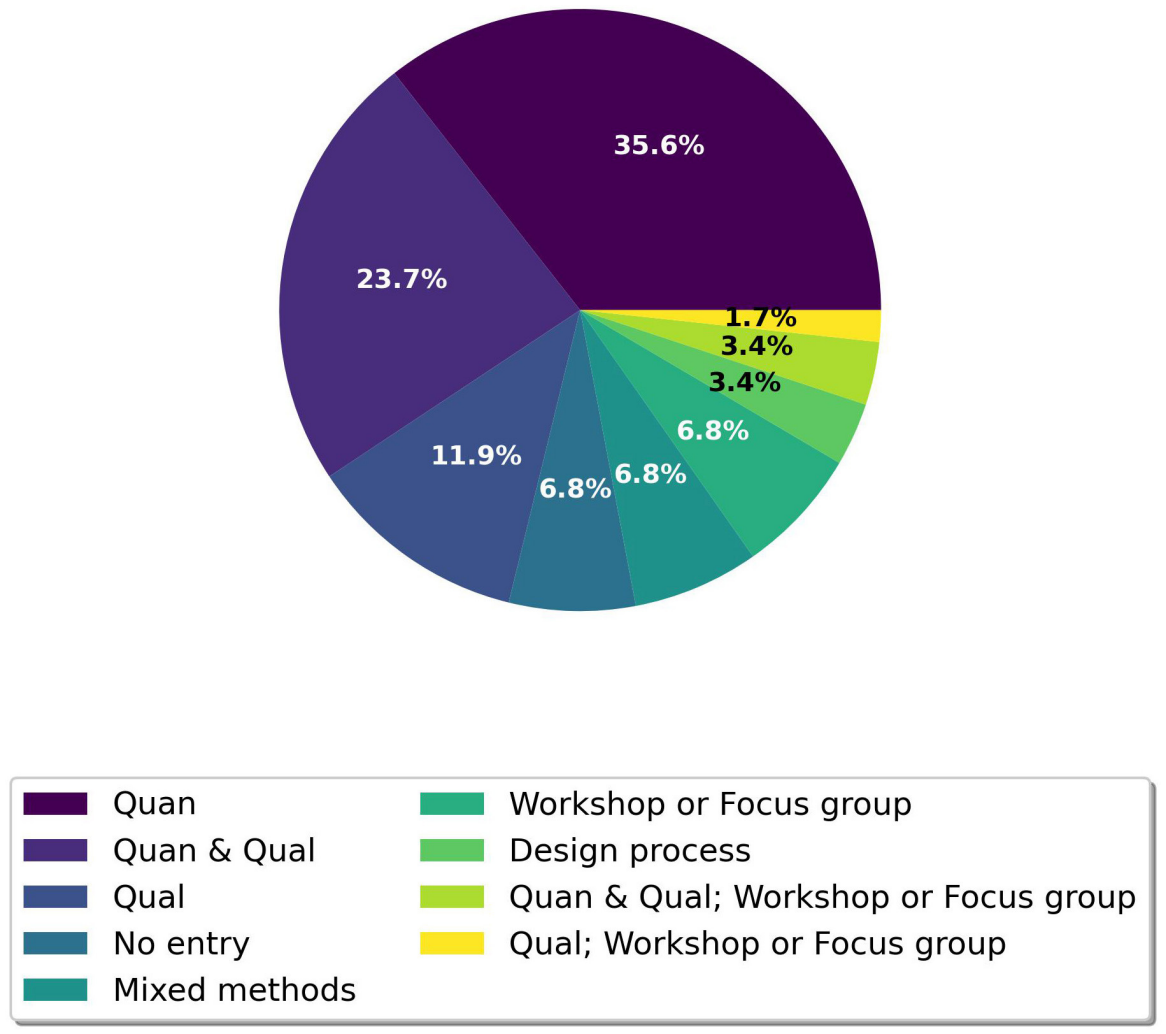
Counts of analytical themes of types of evaluation methods.

The analysis (see Figure 12) revealed that 35.6% (*n* = 21) of the articles relied on *quantitative methods (quan)*, while 11.9% (*n* = 7) used *qualitative approaches (qual)*. A combination of *quantitative and qualitative methods (quan + qual)* was found in 23.7% (*n* = 14) of the articles in which the data were analyzed separately. In contrast, *mixed methods* studies integrated and related the quantitative and qualitative data in 6.8% (*n* = 4). Additionally, two themes emerged that focused on studies that reported *workshops, focus groups*, and *design processes* as outcomes. Specifically, 11.9% (*n* = 7) of the articles incorporated workshops or focus groups, either as standalone studies (*n* = 4), in combination with qualitative methods (*n* = 1), or in conjunction with qualitative and quantitative methods (*n* = 2). Furthermore, 3.4% (*n* = 2) of the studies concentrated on design processes, while 6.8% (*n* = 4) did not specify any evaluation methods, focusing solely on describing their applications.

##### 4.2.4.5 Time-based analysis of evaluation methods

**Figure 13:**
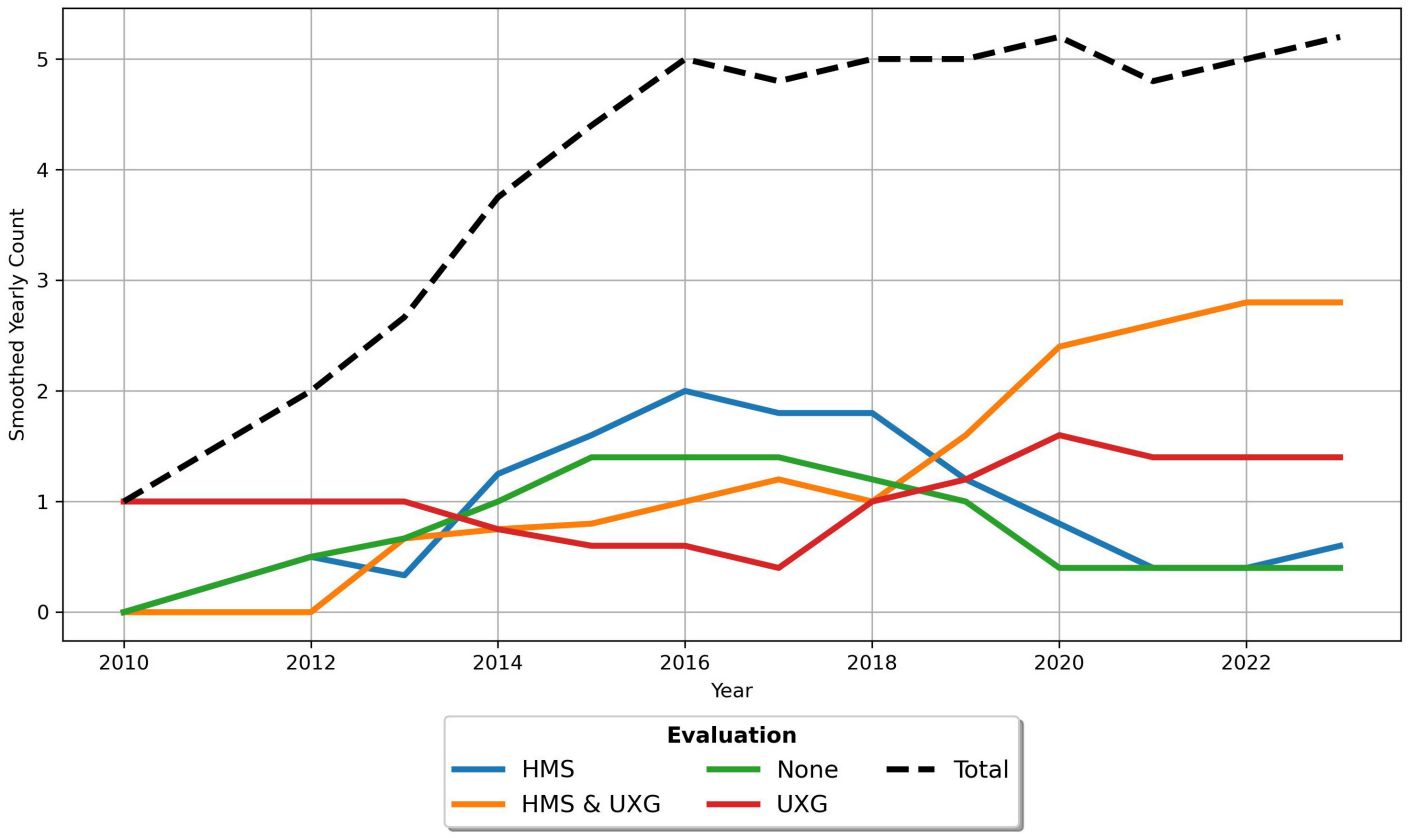
Trend with a rolling mean of 5 for evaluation methods used in articles.

Figure 13 presents a trend chart illustrating the evaluation methods used in the articles. Since 2012, the trend for HMS evaluation methods has steadily increased until 2016, followed by a decline until 2021, when it reached a plateau of approximately 0.5 studies per year. In contrast, the combination of UXG & HMS has demonstrated a consistent upward trajectory since 2012, attaining the status of the most frequently published aspect of evaluation methods by 2019 and reaching a rate of nearly three studies per year by 2023. The number of studies employing UXG aspects alone slightly increased from one to 1.5 per year, surpassing the number of studies utilizing HMS aspects. Notably, the number of studies lacking evaluation methods increased to 1.5 per year by 2014, then steadily declined until 2020, when it reached a plateau.

#### 4.2.5 Interaction of key categories—Objectives, design, type, and evaluation

In this section, we analyze the interaction of the results by using various combinations of the four key categories.The key results show that HMS & UXG aspects are predominantly considered across all categories, except in the evaluation category.

**Figure 14:**
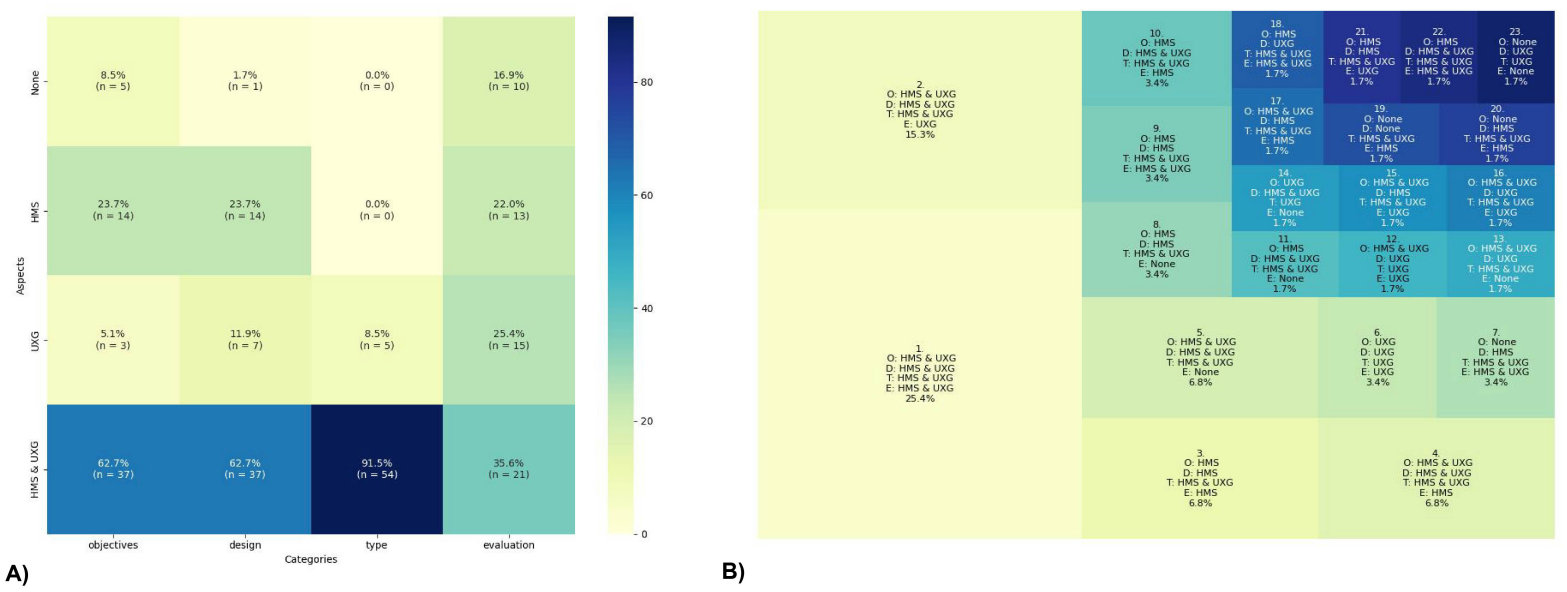
Interaction of the key categories: Objectives (O), Design (D), Types (T), and Evaluation (E). **(A)** shows the heatmap and **(B)** the treemap.

The heatmap (see Figure 14A) illustrates the distribution and interplay of HMS and UXG aspects across the four key categories: *Objectives, Design, Type, and Evaluation*. In the objectives category, HMS aspects concentrate on the specific training objectives, whereas UXG aspects distinguish between human-related and digital training objectives. Design is analyzed from both HMS and UXG aspects, examining the Concepts and Procedures involved in creating interventions. In the type category, the distinction is drawn between HMS aspects, which pertain to the skills and underlying abilities that mitigate the risk of falls, and UXG aspects, which relate to the type of experience offered. Finally, the evaluation methods are categorized according to whether they pertain to UXG, HMS, or shared methods. The analysis reveals that most articles across all categories incorporate both HMS and UXG aspects. Notably, the type category demonstrates the highest degree of overlap, with 91.5% (*n* = 54) of articles addressing both aspects. Conversely, only 5 articles were exclusively labeled as UXG without any HMS classification. In the objectives category, 62.7% (*n* = 37) of the articles define objectives in both HMS and UXG aspects. Additionally, 23.7% (*n* = 14) of the articles focus solely on HMS aspects, while 5.1% (*n* = 3) address solely UXG aspects. Notably, 8.5% (*n* = 5) of the articles did not define any aspect of either category. Similarly, 62.7% (*n* = 37) of the articles address HMS and UXG aspects within the design category. 23.7% (*n* = 14) of the studies in the design category exclusively focus on HMS aspects. Additionally, 11.9% (*n* = 7) of the articles incorporate UXG design aspects, which is slightly higher than the proportion observed for objectives. A single study was found to be lacking any design aspect. The smallest overlap was observed in the evaluation category, where 35.6% (*n* = 21) of the studies included both HMS and UXG aspects. The remaining 38 articles were almost evenly distributed across UXG aspects (25.4%, *n* = 15), HMS aspects (22.0%, *n* = 13), and those with no evaluation aspect at all (17.0%, *n* = 10).

The treemap (see Figure 14B) visually represents the combinations of the four key categories across the aspects. The size of each segment corresponds to the percentage of articles that fall into each combination (*n* = 59). The largest segment in the treemap is characterized by the combination of HMS & UXG aspects across all categories, accounting for 25.4% (*n* = 15) of the articles. Another significant portion, representing 15.3% (*n* = 9) of the articles, includes aspects of UXG & HMS in most categories, with UXG exclusively appearing in the evaluation category. As reflected in the heatmap, in many categories (segments: 1, 2, 3, 4, 5, 6, 8, 9, 19, and 21), which account for 74.7% (*n* = 44) of the articles, the aspects of objectives and design are aligned. Furthermore, a substantial portion of categories (segments: 3, 4, 7, 8, 9, 10, 11, 17, 19, 20, and 22), representing 34.0% (*n* = 20) of the articles, include only HMS or a combination of HMS & UXG aspects, without the sole focus being on UXG. In 39.0% (*n* = 23) of the articles (segments: 1, 3, 6, 10), the objectives and evaluation share the same aspects of disciplines. If a category contains no aspects of HMS or UXG, it is unlikely to have another category labeled as *None* (except for segments 19 and 23). When an article's objectives solely focus on HMS, the other categories often address multiple aspects (segments: 3, 8, 9, 10, 11, 14, 18, 21, and 22). In contrast to the UXG aspects, for which there is a segment that exclusively covers UXG aspects (segment 6), there is no segment that focuses solely on HMS aspects.

## 5 Discussion

**(1) Digital interactive experience- and game-based fall interventions remain a sustained focus of relevance in scientific research**. In recent years, the relevance of digital interactive experience and game-based fall interventions has become increasingly apparent, as evidenced by the continuous publication of studies in this area. Our analysis within this systematic review reveals a high rate of published articles since 2016, illustrating the scientific interest in this field. This trend aligns with the findings of a scoping review conducted in 2012, which also reported an increase in research efforts within the domain of health games over the preceding decade (30). One potential explanation for this sustained focus is the growing necessity for interventions, particularly in light of the rapidly aging global population (1). The WHO has emphasized the importance of developing such interventions (13), further fueling research in this area. Moreover, the increased accessibility and affordability of interactive and extended reality technologies have reduced the obstacles to their creation and deployment (149, 150). Consequently, the intersection between digital technology and game-based interventions continues to be a critical area of study, with significant implications for public health and well-being.

**(2) The potential of UXG in designing digital interactive digital fall prevention interventions remains untapped despite its capacity to address human psychological needs**. Our analysis reveals that nearly one-third of the articles do not include objectives related to UXG. Of the articles that include UXG-related objectives, over 20% focus on training-related objectives. This suggests a missed opportunity, as less than half of the articles leverage UXG's potential to address human needs and their fulfillment as a central objective. Additionally, one-third of the articles reviewed do not incorporate any aspect of UXG into the design, whether conceptually or procedurally. While some studies employ UXG concepts (15.3%), they are relatively uncommon, though still more prevalent than those in HMS (10.2%). Notably, the UXG concepts tend to prioritize human needs, behavior change, and persuasion. In contrast to HMS objectives, the HMS evaluation included psychological factors related to falls, such as fear of falling and fall-related self-efficacy. However, these psychological influences are not defined as objectives in the interventions' design. Instead, research shows that these factors are typically addressed indirectly through motor or motor-cognitive training (151). It is worth mentioning that a few studies use additional fact sheets. UXG offers a unique opportunity to directly address these psychological needs within HMS by embedding them as core objectives within the intervention design. To fully harness the potential of UXG, more attention must be paid to formulating human-related objectives within the intervention. Research also supports the lack of the use of UXG potential, such as the focus on the implementation of accessible user interfaces in motion-based games for older adults (43), or the need to integrate concepts of behavior change in health games (30). UXG should leverage its capabilities to expand beyond procedural implementations or integrate stakeholder preferences. It can also address psychological and human-centered objectives, both independently and in conjunction with HMS-related objectives.

**(3) The collaboration between UXG and HMS has become increasingly important and aligned throughout the study period**. Our analysis of objectives throughout the review period demonstrates that most objectives were formulated from synthesizing aspects of UXG and HMS, with the gap between these and single-aspect objectives steadily widening. Furthermore, the number of single-aspect objectives has declined in both HMS and UXG, indicating a shift toward more integrated objectives. A noteworthy trend since 2010 is the increase in the co-design of interventions encompassing both UXG and HMS aspects, with this trend gaining momentum in recent years. Interestingly, while there was an increase in purely HMS-focused designs from 2014 to 2019, this trend has sharply declined over the past five years. Upon examination of the design of interventions, heatmap analyses reveal that the similar distribution of procedure-based and both concept- and procedure-based design suggests that UXG might influence collaborative work. It is noteworthy that seven articles exclusively show UXG aspects. Concerning evaluation methods, the time-based analysis indicates that studies have increasingly integrated evaluations of both UXG and HMS aspects since 2019, with this trend continuing to increase, while evaluations focusing solely on HMS have decreased markedly. Although there has been a slight increase in UXG-only evaluations, the prevailing trend is toward more comprehensive assessments incorporating both aspects. Collaborative aspects are predominant within the four key categories under study, with a quarter of all items pertaining to collaboration representing the largest proportion across all categories. Nevertheless, nearly 75% of the studies are still not fully integrated, highlighting a significant avenue for future improvement. This topic is inherently cross-disciplinary, with UXG and HMS emerging as the most prominent aspects across all four key categories. Therefore, given their intrinsic interconnectivity, future research should prioritize reporting on all four key categories. This comprehensive reporting will facilitate a more nuanced understanding of study outcomes by establishing direct connections to the application components, design and development, and objectives, ultimately enabling reproducibility and continuous improvement in future research.

**(4) Clear and consistent terminology within UXG and HMS aspects of digital interactive fall interventions is lacking, undermining their transparency, reproducibility, and potential for refinement**. In UXG, there is a noticeable inconsistency in the terminology used to describe game-related design procedures such as gamification and game design, showing a low specialized term depth. The network analysis shows that different procedures are often strongly interconnected, with terms such as exergame, gamification, and game often occurring together in the same articles. This pattern aligns with findings from UXG research, which identified a lack of consistent terminology (69). For example, the terms gamification and game design are clearly distinguished from each other, particularly concerning their processes and implementation (68, 70, 71). However, our analysis indicates that while these procedures are frequently referenced, their actual design details are often absent, suggesting the need for more detailed descriptions. Similarly, the descriptions of the exercises included in the interventions in HMS vary considerably in terms of the level of detail provided. While nearly 90% of the articles include a basic description or general labeling of exercises used, all studies lack a report of how exercises were integrated into a specific training plan within the application, necessary for accurate replication or assessment. This general lack of detailed description in both UXG processes and HMS exercises highlights the need for standardized guidelines and a thesaurus to ensure that interventions are described thoroughly and consistently. Establishing clear patterns and providing guidance for documentation will facilitate the comprehensive coverage of all aspects of fall prevention exercises, experiences, and technology types.

**(5) The application of concept-based design in UXG and HMS aspects is gaining prominence to foster collaboration and enhance mutual understanding among researchers**. However, the current application of concepts in these interventions remains limited. Our findings indicate that only around 10% of the reviewed articles incorporate an HMS concept, while around 15% use a UXG concept. Moreover, there is no consistent use of concepts within HMS. Each concept appears only once or twice across all studies, highlighting a lack of standardization and widespread adoption. Despite these limitations, there is a promising trend toward greater use of concepts. Since 2016, there has been a notable decline in studies that do not employ any concepts, indicating a growing appreciation for their value. In articles where both UXG and HMS contribute to a study, a concept drawn from one of the two areas is employed in around 17% of these cases. This suggests that cross-disciplinary collaboration is associated with a higher probability of concept integration. The relevance of concept use is also substantiated by research, showing that incorporating theoretical frameworks is vital in advancing health games yet is currently undervalued in research (30). Emphasizing concept-based approaches will enhance understanding and collaboration and provide a framework for other researchers to build upon, thereby promoting transparency, reproducibility, and further development in these fields.

**(6) The procedure-based design in UXG and HMS aspects differs strongly, and there is a compelling argument for aligning these to enhance the overall quality of interventions**. In HMS, the procedures are predominantly research-driven, emphasizing using the outcomes and findings from previous studies as the foundation for designing interventions. This includes using concrete principles, strategies, and concepts for intervention design, which, in the present articles, is focused on the augmentation of exercise difficulty and the provision of feedback. HMS procedures also incorporate evidence-based fall prevention programs and research-based training, drawing heavily on existing data and interventions. In contrast, UXG procedures are process-driven and heavily centered on human experience. The most commonly used approach is human-centered design (*n* = 33), while participatory design and co-design are also widely used (*n* = 15). However, there is a notable gap in applying these processes to the design of fall interventions, highlighting a lack of processes specified for translating fall intervention exercises into design.

**(7) A notable discrepancy exists in the use of shared evaluation methods and the interplay of results between HMS and UXG aspects**. HMS predominantly relies on quantitative methods, with approximately 75% of evaluations based on measurements. In contrast, UXG employs a blend of qualitative and quantitative methods, often focusing on assessing user experience. Although two-thirds of study objectives integrate both HMS and UXG aspects, only approximately one-third of the articles employ shared evaluation methods. Since 2019, there has been a positive trend toward using both HMS and UXG evaluation methods, underscoring the increasing relevance of shared assessments. However, most studies still favor quantitative methods (around 35%) or a combination of quantitative and qualitative methods (over 20%), often without fully integrating the results. Only approximately 7% of articles employ mixed methods that explore the interplay between outcomes. This highlights the need for more shared evaluation methods that can effectively relate and combine the findings from both aspects, enabling a more comprehensive understanding of how HMS and UXG objectives interact and inform each another.

**(8) The potential to address various aspects of fall prevention appears to be untapped, as most interventions are primarily designed to enhance motor skills, with a predominant focus on single-task activities**. This systematic review reveals that nearly half of the HMS objectives are exclusively focused on motor skills and abilities addressed through specific exercises within the application. This focus is evident in that two-thirds of the articles emphasize enhancing motor skills, particularly in mobility, gait, and physiological parameters. Regarding the nature of tasks employed in these articles, nearly 40% concentrate exclusively on single-task activities. This is followed by studies that incorporate both single- and dual-tasks, accounting for almost 30 %, while only a little over 20 % of the studies focus solely on dual-tasks. This emphasis on single-task interventions is notable, although research indicates the potential benefits of dual-task training in fall prevention (16). A focus on single tasks alone does not fully realize the potential of digital interactive applications, particularly those involving games that are inherently suited to promoting dual-tasks (74, 152). The full potential of these digital interventions can be harnessed by integrating all natures of tasks especially dual-task training and remediating interference, thereby providing versatile fall interventions that are key to prevention (3).

**(9) The increasing prevalence of immersive technological enablers presents novel opportunities to enhance training in fall prevention**. Currently, most articles concentrate on enhancing the motor skills of stand and mobility, with only a limited number addressing gait. Of the technologies examined, screen-based VR, a low-immersion technology, was the most widely frequently used, appearing in nearly 50% of the articles. This dominance of publications using non-immersive technologies aligns with other research findings (153, 154). However, its utilization has exhibited a gradual decline since 2016. In contrast, there has been a notable increase in the use of immersive technologies, especially MR, while research into fully immersive VR and AR is also beginning to gain momentum. Fully immersive VR presents specific challenges, such as the inability to perceive obstacles in the real world or the potential for motion sickness, which may increase the risk of falls (155). Nevertheless, within controlled environments, it offers unique opportunities to simulate authentic, real-world scenarios, such as hiking in the mountains or negotiating congested urban routes. This can improve quality of life and be beneficial for healthy older adults, as it allows them to practice real-world tasks in a safe, immersive environment (72, 156). Unexpectedly, AR is not more widely used in this analysis, given its potential to enhance real-world environments with digital elements and thereby facilitate fall prevention in everyday situations. AR could enrich real-world scenarios by providing additional support, motivation, feedback, entertainment, and other needs-based enhancements, making it a powerful tool for training “as before” in familiar environments (153). It is essential to consider that technologies should be selected according to the target group's specific needs and the intervention's objectives as they use the vast space of possibilities. By leveraging different technologies, a broader spectrum of motor and cognitive skills, as well as human needs relevant to fall prevention can be effectively targeted and trained in a safe environment without the consequences of real world falling.

**(10) While collaboration is already established, interdisciplinary collaboration should be actively encouraged and expanded**. However, the overall quality of articles in this area still requires improvement. Our analysis shows that beside that just around 15 % identified themselves as cross-disciplinary, most aspects of the articles incorporated both HMS and UXG across all four key categories: objectives (62.7%, *n* = 37), design and development (62.7%, *n* = 37), types of interventions (91.5%, *n* = 54), and evaluation methods (35.6%, *n* = 21). Notably, the objectives for design and development were determined mainly by combining HMS and UXG rather than by one of the two aspects alone. However, we anticipated a higher number of joint objectives that would fully leverage the strengths of HMS and UXG. If the primary goal of the articles had been to translate fall prevention training into a digital interactive application, we would have expected more training concepts specifically developed for digitization or game-based integration. Given the procedural nature of the articles, it would have been reasonable to expect a greater emphasis on developing HMS and UXG objectives to maximize the potential of combining effective training with creating positive and meaningful experiences. The analysis of design and development revealed a notable scarcity of concept-based approaches, with most articles relying solely on procedures. Furthermore, descriptions of digital interactive fall interventions frequently lacked specificity, particularly concerning experience-related terminology and exercise descriptions. While evaluation methods increasingly drew from both HMS and UXG aspects, only four studies employed a mixed-methods approach to explore the interplay between these methods. It is imperative to emphasize the reporting across all four key categories, as they are profoundly interconnected and naturally lend themselves to interdisciplinary work. There is a clear need for holistic concepts and procedures that support cross-disciplinary and, in particular, interdisciplinary collaboration. This assertion is supported by the findings of the scoping review conducted by Kharrazi et al. (30) which pertains to the domain of health games. Developing a shared thesaurus and standardized guidelines would greatly streamline this process, ensuring clarity and consistency in reporting. Incorporating supplementary reporting guidelines could assist researchers in succinctly encapsulating their work, particularly given the constrained space often available in publications.

## 6 Limitations

This systematic review was constrained by some limitations, primarily due to the considerable heterogeneity of the articles included. The inclusion of various types of articles and study designs introduced a high degree of heterogeneity, which rendered detailed analysis challenging due to the absence of a standardized thesaurus and consistent definitions. This resulted in a notable challenge in data collection at a uniform level of abstraction. Another limitation is that we did not assess the quality of the selected studies using standardized instruments such as the Mixed Methods Appraisal Tool (MMAT) (157). While we intentionally chose not to perform a quality assessment for this review, it remains an important step for future efficacy assessments. Conducting such an evaluation in conjunction with the findings of this review would be beneficial. Additionally, the categorization of the studies may have been influenced by potential researcher bias. Furthermore, each article was evaluated independently not as part of broader research projects, which could have provided additional insights for this review. Finally, this review does not provide detailed information on specific objectives, design approaches, types of interventions, or evaluation methods used in the studies.

## 7 Conclusion

This systematic review was conducted cross-disciplinary to analyze the current state of digital interactive experience- and game-based fall interventions for community-dwelling healthy older adults. A qualitative thematic synthesis was performed to identify how the aspects of Human Movement Science (HMS) as well as User Experience and Game Design (UXG) are integrated across the various fields of study on this topic. Four key categories were identified for this purpose: Objectives of Design and Development, Design and Development, Types of Intervention, and Evaluation Methods. The review found that collaboration already plays a pivotal role, with 25.4% of the articles addressing both HMS and UXG aspects across all key categories. However, there is a lack of guidance in the form of a common thesaurus for precise terminology and shared reporting standards. Holistic concepts and procedures must be established to guide common design processes and improve the overall quality of studies. The potential of UXG has yet to be fully realized, particularly in designing interventions that address psychological human needs. Furthermore, the review identified a lack of common evaluation methods that could facilitate the integration of the results of both HMS and UXG aspects. Interdisciplinary collaboration should be further encouraged to enhance the quality and impact of future research. Further work should analyze how integrating both HMS and UXG aspects influences the effectiveness of studies. Moreover, a more detailed examination of the specific objectives, design methods, evaluation techniques, as well as the specific exercises and technological setups used may provide additional valuable insights.

## Data Availability

The original contributions presented in the study are included in the article/Supplementary material, further inquiries can be directed to the corresponding author.

## Supplementary material

Data Sheet 1 Data Synthesis

Data Sheet 2 Search Matrices

Data Sheet 3 Search Strings

Data Sheet 4 PRISMA checklist
