# Supplementary material for: Digital interactive experience- and game-based fall interventions for community-dwelling healthy older adults: a cross-disciplinary systematic review
Source: Front Public Health. 2025 Jan 23;12:1489258. doi: 10.3389/fpubh.2024.1489258 (PMC11799000; doi:10.3389/fpubh.2024.1489258)
Supplement: Data Sheet 1 — Data Synthesis [file Data_Sheet_1.pdf]

Author_Year	Title
Garcia et al. (2018)	assessing user engagement with a fall prevention game as an unsupervised exercise program for older people
Garcia et al. (2016)	a bespoke kinect stepping exergame for improving physical and cognitive function in older people: a pilot study
Bakker et al. (2020)	balance training monitoring and individual response during unstable vs. stable balance exergaming in elderly adults findings from a randomized controlled trial
Morat et al. (2019)	effects of stepping exergames under stable versus unstable conditions on balance and strength in healthy community-dwelling older adults: a three-armed randomized controlled trial
Gallou-Guyot et al. (2023)	feasibility and potential cognitive impact of a cognitive-motor dual-task training program using a custom exergame in older adults a pilot study
Gschwind et al. (2015)	ict-based system to predict and prevent falls istoppfalls results from an international multicenter randomized controlled trial
Vaziri et al. (2016)	exploring user experience and technology acceptance for a fall prevention system results from a randomized clinical trial and a living lab
Marston et al. (2015)	the design of a purpose-built exergame for fall prediction and prevention for older people
Sato et al. (2015)	improving walking, muscle strength, and balance in the elderly with an exergame using kinect a randomized controlled trial
Sato et al. (2014)	s
Tan et al. (2022)	effectiveness and usability of the system for assessment and intervention of frailty for community-dwelling pre-frail older adults a pilot study
Zeng et al. (2019)	saif: a system for long-term assessment and intervention of frailty with gamification
Delbaere et al. (2021)	e-health standingtall balance exercise for fall prevention in older people results of a two year randomised controlled trial
Ambrens et al. (2023)	exploring older adults experiences of a home-based, technology-driven balance training exercise program designed to reduce fall risk a qualitative research study within a randomized controlled trial
Valenzuela et al. (2018b)	an interactive home-based cognitive-motor step training program to reduce fall risk in older adults qualitative descriptive study of older adults experiences and requirements
Schoene et al. (2015)	interactive cognitive-motor step training improves cognitive risk factors of falling in older adults – a randomized controlled trial
Smith et al. (2013)	step training system: an ict solution to measure and reduce fall risk in older adults
Schoene et al. (2013)	a randomized controlled pilot study of home-based step training in older people using videogame technology
Uzor and Baillie (2019)	recov-r evaluation of a home-based tailored exergame system to reduce fall risk in seniors
Uzor et al. (2012)	senior designers empowering seniors to design enjoyable falls rehabilitation tools
Uzor and Baillie (2013)	exploring & designing tools to enhance falls rehabilitation in the home
Uzor and Baillie (2014)	investigating the long-term use of exergames in the home with elderly fallers
Adcock et al. (2019)	a pilot study of an in-home multicomponent exergame training for older adults feasibility, usability and pre-post evaluation
Adcock et al. (2020)	a usability study of a multicomponent video game-based training for older adults
Whyatt et al. (2015)	a wii bit of fun a novel platform to deliver effective balance training to older adults
Merriman et al. (2018)	acceptability of a custom-designed game, cityquest, aimed at improving balance confidence and spatial cognition in fall-prone and healthy older adults
Nishchyk et al. (2020)	an augmented reality game for helping elderly to perform physical exercises at home
Kamnardsiri et al. (2021)	an interactive physical-cognitive game-based training system using kinect for older adults development and usability study
Dewick and Stanmore (2017)	applying game thinking to slips, trips and falls prevention
Mostajeran et al. (2020)	augmented reality for older adults exploring acceptability of virtual coaches for home-based balance training in an aging population
Chen et al. (2020)	augmented reality-assisted training with selected tai-chi movements improves balance control and increases lower limb muscle strength in older adults a prospective randomized trial
Brito et al. (2015)	balance assessment in fall-prevention oriented exergames
Doyle et al. (2010)	base - an interactive technology solution to deliver balance and strength exercises to older adults
Pedroli et al. (2018)	characteristics, usability, and users experience of a system combining cognitive and physical therapy in a virtual environment positive bike
Guimaraes et al. (2018)	design and evaluation of an exergame for motor-cognitive training and fall prevention in older adults
Velazquez et al. (2013)	design of exergames with the collaborative participation of older adults
Chartomatsidis and Goumopoulos (2020)	development and evaluation of a motion-based exercise game for balance improvement
Willaert et al. (2020)	does a novel exergame challenge balance and activate muscles more than existing off-the-shelf exergames?
Kayama et al. (2013)	effect of a kinect-based exercise game on improving executive cognitive performance in community-dwelling elderly
Kim and Xiong (2022)	effectiveness and usability of a novel kinect-based tailored interactive fall intervention system for fall prevention in older people a preliminary study
van Diest et al. (2016)	exergames for unsupervised balance training at home a pilot study in healthy older adults
Goumopoulos and Koumanakos (2023)	exergaming with the game2awe platform design, implementation and evaluation insights
Papi et al. (2020)	feasibility and acceptability study on the use of a smartphone application to facilitate balance training in the ageing population
Ogonowski et al. (2016)	ict-based fall prevention system for older adults qualitative results from a long-term field study
Schwenk et al. (2014)	interactive balance training integrating sensor-based visual feedback of movement performance a pilot study in older adults
Munoz et al. (2019)	lessons learned from gamifying functional fitness training through human-centered design methods in older adults
Chen et al. (2012)	lower limb power rehabilitation llpr using interactive video game for improvement of balance function in older people
Janols et al. (2022)	older adults as designers of behavior change strategies to increase physical activity-report of a participatory design process
Seinsche et al. (2023)	older adults needs and requirements for a comprehensive exergame-based telerehabilitation system a focus group study
Ogawa et al. (2019)	physiological responses and enjoyment of kinect-based exergames in older adults at risk for falls a feasibility study
Daniels et al. (2023)	promoting physical activity and a healthy active lifestyle in community-dwelling older adults a design thinking approach for the development of a mobile health application
Garcia and Felix Navarro (2015)	stepkinnection a fall prevention game mindfully designed for the elderly
Retz et al. (2023a)	towards co-creative interdisciplinary exergame design processes a theory-based approach of a vr-exergame fall prevention training
Hsieh et al. (2014)	virtual reality system based on kinect for the elderly in fall prevention
Gaggioli et al. (2017)	“positive bike” – an immersive biking experience for combined physical and cognitive training of elderly patients
Sandlund et al. (2016)	towards a mobile exercise application to prevent falls: a participatory design process
Pettersson et al. (2019)	‘managing pieces of a personal puzzle’ — older people’s experiences of self-management falls prevention exercise guided by a digital program or a booklet
Lindgren et al. (2014)	end users transforming experiences into formal information and process models for personalised health interventions
Meurer and Wieching (2012)	motivating elderly people to use fall preventive exercise training games at home: are community based ict features always a good choice?

Crossdisciplinary	Objective_HMS_Category	Objective_UXG_Category
no	motor	Human and digital training related
no	motor; cognitive	Digital training related
no	motor; cognitive	no entry
no	motor; cognitive	no entry
yes; (interdisciplinary as one affilitation)	motor; cognitive	Human and digital training related
no	No entry	no entry
no	motor; interference	no entry
no	motor; interference	no entry
no	No entry	no entry
no	No entry	no entry
no	motor	Human and digital training related
no; (word multidisciplinary in context of frailty, not disciplines)	motor	Human and digital training related
no	motor	no entry
no	motor	Digital training related
no	motor; cognitive; interference	Human related
no	motor; cognitive; interference	no entry
no	motor; cognitive	Digital training related
no	motor; cognitive	Digital training related
no	motor	Human and digital training related
no	motor	Human and digital training related
no	motor	Digital training related
no	motor	Digital training related
no	motor; cognitive; interference	Human and digital training related
no	motor; cognitive; interference	Digital training related
no	motor	Human related
no	motor; cognitive; interference	Human related
no	motor	Human and digital training related
no	motor; cognitive; interference	Human and digital training related
yes; (multidisciplinary)	motor	Human related
no	motor; cognitive	Digital training related
no	motor	no entry
no	motor	no entry
yes; (multidisciplinary)	motor	Human and digital training related
no	motor; cognitive; interference	Human related
no	motor; cognitive; interference	Digital training related
no	No entry	Human and digital training related
no	motor	Human related
no	motor	Human related
no	motor; cognitive; interference	no entry
no	No entry	no entry
no	motor	no entry
no; (multidisciplinary focus group as method)	motor; cognitive	Digital training related
no	motor	no entry
no	motor	Human related
yes; (interdisciplinary as one affilitation)	motor	Digital training related
yes; (interdisciplinary, multidisciplinary)	motor	Digital training related
no	motor	no entry
no	motor	Human related
no	motor; cognitive	Human related
no	motor; cognitive; interference	no entry
no	No entry	Human and digital training related
no	motor; cognitive	Human and digital training related
yes	interference	Human related
no	motor	Human related
no	motor; cognitive; interference	no entry
yes; (cross-disciplinary)	motor	Human related
yes; (multidisciplinary)	motor	Human related
no	No entry	no entry
no	No entry	Human related

Design and Developments_HMS_Procedure_Category	Design and Development_HMS_Concept
concrete principles, strategies and concepts; research based fall prevention / training / recommendations / guidelines	-
concrete principles, strategies and concepts; research based fall prevention / training / recommendations / guidelines	-
concrete principles, strategies and concepts; research based fall prevention / training / recommendations / guidelines	-
research based fall prevention / training / recommendations / guidelines	-
concrete principles, strategies and concepts; research based fall prevention / training / recommendations / guidelines	-
evidence based fall programms; research based fall prevention / training / recommendations / guidelines	-
concrete principles, strategies and concepts; evidence based fall programms; research based fall prevention / training / recommendations / guidelines	-
concrete principles, strategies and concepts; evidence based fall programms; research based fall prevention / training / recommendations / guidelines	-
concrete principles, strategies and concepts	-
-	-
human input; research based fall prevention / training / recommendations / guidelines	-
research based fall prevention / training / recommendations / guidelines	-
-	-
-	-
concrete principles, strategies and concepts	-
concrete principles, strategies and concepts; tests / scales	-
concrete principles, strategies and concepts; tests / scales	-
concrete principles, strategies and concepts; tests / scales	-
concrete principles, strategies and concepts; evidence based fall programms	-
evidence based fall programms	-
concrete principles, strategies and concepts; evidence based fall programms	-
concrete principles, strategies and concepts; evidence based fall programms	-
concrete principles, strategies and concepts; research based fall prevention / training / recommendations / guidelines	framework for design and evaluation of complex interventions to improve health (Campbell)
concrete principles, strategies and concepts; research based fall prevention / training / recommendations / guidelines	framework for design and evaluation of complex interventions to improve health (Campbell)
concrete principles, strategies and concepts	-
concrete principles, strategies and concepts; research based fall prevention / training / recommendations / guidelines	-
research based fall prevention / training / recommendations / guidelines	-
concrete principles, strategies and concepts; research based fall prevention / training / recommendations / guidelines	-
evidence based fall programms; research based fall prevention / training / recommendations / guidelines	-
concrete principles, strategies and concepts; research based fall prevention / training / recommendations / guidelines	-
research based fall prevention / training / recommendations / guidelines	-
concrete principles, strategies and concepts; evidence based fall programms; tests / scales	-
concrete principles, strategies and concepts; evidence based fall programms	-
concrete principles, strategies and concepts	Wickens' theory of shared attentional resources; bottleneck hypothesis
concrete principles, strategies and concepts; research based fall prevention / training / recommendations / guidelines	-
Iterative task design; human input; research based fall prevention / training / recommendations / guidelines	dual flow model
human input	-
research based fall prevention / training / recommendations / guidelines	-
concrete principles, strategies and concepts	-
concrete principles, strategies and concepts; human input	-
research based fall prevention / training / recommendations / guidelines	-
concrete principles, strategies and concepts; research based fall prevention / training / recommendations / guidelines	-
concrete principles, strategies and concepts; research based fall prevention / training / recommendations / guidelines; tests / scales	-
concrete principles, strategies and concepts; evidence based fall programms; research based fall prevention / training / recommendations / guidelines	-
research based fall prevention / training / recommendations / guidelines	-
research based fall prevention / training / recommendations / guidelines	FITT (frequency, intensity, time, type) model
research based fall prevention / training / recommendations / guidelines	-
-	-
-	-
concrete principles, strategies and concepts; research based fall prevention / training / recommendations / guidelines	-
-	-
concrete principles, strategies and concepts; research based fall prevention / training / recommendations / guidelines	-
concrete principles, strategies and concepts	Gentile's Taxonomy of Motor Skills
concrete principles, strategies and concepts; tests / scales	-
concrete principles, strategies and concepts	-
evidence based fall programms; human input	-
evidence based fall programms	-
-	-
-	-

Design and Development_HMS_Design and Development	Design and Development_UXG_Procedure_Category	Design and Development_UXG_Concept_Category
Procedure based	game design practice; gamification; human centered design	-
Procedure based	game design practice; gamification; human centered design	-
Procedure based	gamification	-
Procedure based	-	-
Procedure based	gamification; general recommendations and guidelines; human centered design	-
Procedure based	-	-
Procedure based	-	-
Procedure based	-	-
Procedure based	-	-
Not Procedure or Concept based	-	-
Procedure based	gamification; human centered design	Human Needs and Embodiment
Procedure based	game design practice; gamification; human centered design	-
Not Procedure or Concept based	behaviour change techniques / persuasive design; human centered design	-
Not Procedure or Concept based	behaviour change techniques / persuasive design; human centered design	-
Procedure based	modified existing games	-
Procedure based	gamification; modified existing games	-
Procedure based	modified existing games	-
Procedure based	modified existing games	-
Procedure based	human centered design; participatory design / co-design	-
Procedure based	human centered design; participatory design / co-design	-
Procedure based	human centered design	-
Procedure based	game design practice; participatory design / co-design	-
Procedure and Concept based	game design practice; human centered design	-
Procedure and Concept based	game design practice; human centered design	-
Procedure based	-	-
Procedure based	gamification	-
Procedure based	gamification; human centered design	-
Procedure based	game design practice; human centered design	-
Procedure based	game design practice; gamification; general recommendations and guidelines; human centered design; participatory design / co-design	Behavior Change and Persuasion; Human Needs and Embodiment
Procedure based	human centered design	-
Procedure based	-	-
Procedure based	gamification	-
Procedure based	human centered design	-
Procedure and Concept based	-	-
Procedure based	game design practice; human centered design; modified existing games	-
Procedure and Concept based	human centered design; participatory design / co-design	Flow Models; Gesture Design Based on Theoretics
Procedure based	general recommendations and guidelines; human centered design	-
Procedure based	human centered design	-
Procedure based	modified existing games	-
Procedure based	-	-
Procedure based	-	-
Procedure based	game design practice; human centered design; participatory design / co-design	Flow Models
Procedure based	-	-
Procedure based	behaviour change techniques / persuasive design; human centered design; participatory design / co-design	Human Needs and Embodiment
Procedure based	human centered design	-
Procedure and Concept based	game design practice; general recommendations and guidelines; human centered design; participatory design / co-design	-
Procedure based	-	-
Not Procedure or Concept based	human centered design; participatory design / co-design	Behavior Change and Persuasion; Human Needs and Embodiment
Not Procedure or Concept based	human centered design	-
Procedure based	-	-
Not Procedure or Concept based	human centered design; participatory design / co-design	Behavior Change and Persuasion
Procedure based	game design practice; gamification; general recommendations and guidelines; human centered design	-
Procedure and Concept based	game design practice; human centered design; participatory design / co-design	Game Structure; Human Needs and Embodiment
Procedure based	gamification	-
Procedure based	-	-
Procedure based	human centered design; participatory design / co-design	-
Procedure based	participatory design / co-design	Behavior Change and Persuasion
Not Procedure or Concept based	human centered design; participatory design / co-design	-
Not Procedure or Concept based	behaviour change techniques / persuasive design; gamification; human centered design; participatory design / co-design	-

Design and Development_UXG_Design and Development	Type_HMS_Category	Type_HMS_Category_dual_task	Type_UXG_Category
Procedure based	Cognition; Mobility: dynamic postural control	single task; dual task	game-related
Procedure based	Cognition; Mobility: dynamic postural control	single task; dual task	game-related
Procedure based	Cognition; Mobility: dynamic postural control	single task; dual task	game-related
Not Procedure or Concept based	Cognition; Mobility: dynamic postural control	single task; dual task	game-related
Procedure based	Cognition; Mobility: dynamic postural control; Physiological Parameters	single task; dual task	game-related
Not Procedure or Concept based	Cognition; Mobility: dynamic postural control; Physiological Parameters; Stand: static postural control	single task; dual task	game-related
Not Procedure or Concept based	Cognition; Mobility: dynamic postural control; Other; Physiological Parameters; Stand: static postural control	single task; dual task	game-related
Not Procedure or Concept based	Cognition; Mobility: dynamic postural control; Other; Physiological Parameters; Stand: static postural control	single task; dual task	game-related
Not Procedure or Concept based	Physiological Parameters; Stand: static postural control	single task	game-related
Not Procedure or Concept based	Physiological Parameters; Stand: static postural control	single task	game-related
Procedure and Concept based	Mobility: dynamic postural control; Other; Physiological Parameters	single task	game-related
Procedure based	Mobility: dynamic postural control; Other; Physiological Parameters	single task	game-related
Procedure based	Gait: dynamic postural control; Mobility: dynamic postural control; Other; Stand: static postural control	single task	not game-related
Procedure based	Gait: dynamic postural control; Mobility: dynamic postural control; Other; Stand: static postural control	single task	not game-related
Procedure based	Cognition; Mobility: dynamic postural control	single task; dual task	game-related
Procedure based	Cognition; Mobility: dynamic postural control	single task; dual task	game-related
Procedure based	Cognition; Mobility: dynamic postural control	dual task	game-related
Procedure based	Cognition; Mobility: dynamic postural control	dual task	game-related
Procedure based	Mobility: dynamic postural control; Physiological Parameters; Stand: static postural control	single task	game-related
Procedure based	Physiological Parameters; Stand: static postural control	single task	game-related
Procedure based	Mobility: dynamic postural control; Physiological Parameters; Stand: static postural control	single task	game-related
Procedure based	Mobility: dynamic postural control; Physiological Parameters; Stand: static postural control	single task	game-related
Procedure based	Cognition; Mobility: dynamic postural control; Physiological Parameters; Stand: static postural control	single task; dual task	game-related
Procedure based	Cognition; Mobility: dynamic postural control; Physiological Parameters	single task; dual task	game-related
Not Procedure or Concept based	Cognition; Stand: static postural control	single task; dual task	game-related
Procedure based	Cognition; Stand: static postural control	dual task	game-related
Procedure based	Physiological Parameters	single task	game-related
Procedure based	Cognition; Mobility: dynamic postural control; Physiological Parameters; Stand: static postural control	dual task	game-related
Procedure and Concept based	Other; Physiological Parameters	single task	game-related
Procedure based	Cognition; Gait: dynamic postural control; Stand: static postural control	single task	game-related
Not Procedure or Concept based	Mobility: dynamic postural control	single task	not game-related
Procedure based	Stand: static postural control	single task	game-related
Procedure based	Stand: static postural control	single task	not game-related
Not Procedure or Concept based	Cognition; Other	dual task	game-related
Procedure based	Cognition; Mobility: dynamic postural control	dual task	game-related
Procedure and Concept based	No entry	No entry	game-related
Procedure based	Cognition; Stand: static postural control	single task	game-related
Procedure based	Physiological Parameters; Stand: static postural control	single task; dual task	game-related
Procedure based	Mobility: dynamic postural control	dual task	game-related
Not Procedure or Concept based	Cognition; Gait: dynamic postural control; Other; Physiological Parameters; Stand: static postural control	single task	game-related
Not Procedure or Concept based	Stand: static postural control	single task	game-related
Procedure and Concept based	Cognition; Mobility: dynamic postural control	single task; dual task	game-related
Not Procedure or Concept based	Mobility: dynamic postural control; Physiological Parameters; Stand: static postural control	dual task	not game-related
Procedure and Concept based	Mobility: dynamic postural control; Physiological Parameters; Stand: static postural control	single task; dual task	game-related
Procedure based	Stand: static postural control	single task	game-related
Procedure based	Mobility: dynamic postural control; Stand: static postural control	single task	game-related
Not Procedure or Concept based	Physiological Parameters	single task	game-related
Procedure and Concept based	Other; Physiological Parameters	No entry	not game-related
Procedure based	No entry	single task; dual task	game-related
Not Procedure or Concept based	Cognition; Mobility: dynamic postural control; Stand: static postural control	dual task	game-related
Procedure and Concept based	No entry	No entry	not game-related
Procedure based	Cognition; Mobility: dynamic postural control	single task; dual task	game-related
Procedure and Concept based	Cognition; Gait: dynamic postural control	dual task	game-related
Procedure based	Mobility: dynamic postural control	single task	not game-related
Not Procedure or Concept based	Cognition; Other	dual task	not game-related
Procedure based	Other; Physiological Parameters	No entry	not game-related
Procedure and Concept based	Gait: dynamic postural control; Other; Physiological Parameters; Stand: static postural control	No entry	not game-related
Procedure based	No entry	No entry	not game-related
Procedure based	No entry	No entry	game-related

Type_of_game_Category	Type_Technology_Category
game; interactive; system	VR (screen based)
exergame; game; gamification; program	VR (screen based)
exergame; exergame based; game	MR
exergame; exergame based; game	MR
exergame; game; gamification; interactive	MR
Information and Communication Technology / Platform; exergame; program; system; technology(-enabled / based)	VR (screen based)
Information and Communication Technology / Platform; exergame; system; technology(-enabled / based)	VR (screen based)
Information and Communication Technology / Platform; exergame; system; technology(-enabled / based)	VR (screen based)
exergame; game	VR (screen based)
exergame; game	VR (screen based)
gamification; system; technology(-enabled / based)	MR
serious game; system	MR
e-health / m-health; program	touch-based interaction
e-health / m-health; program	touch-based interaction
exergame; interactive; program; system; technology(-enabled / based)	MR
exergame; game; interactive; system	MR
exergame; game; system	MR
exergame; game; technology(-enabled / based)	MR
exergame; system	VR (screen based)
game	VR (screen based)
game; game based	VR (screen based)
exergame; game	VR (screen based)
exergame	VR (screen based)
exergame	VR (screen based)
game; game based; interactive; system	MR
game; serious game	MR
exergame; game	VR (screen based)
game based; interactive; system	MR
application; gamification	touch-based interaction
exergame; game; gamification; system	AR
system	VR (screen based)
exergame; game	MR
interactive; program; system; technology(-enabled / based)	VR (screen based)
application; game	MR
exergame; exergame based; game; interactive	VR (screen based)
exergame	-
exergame; game	VR (screen based)
exergame; game	VR (screen based)
game	VR (screen based)
exergame; interactive; system	VR (screen based)
exergame; game	VR (screen based)
exergame; game; game based	MR; VR (screen based); VR (fully immersive)
application; technology(-enabled / based)	touch-based interaction
Information and Communication Technology / Platform; exergame; system; technology(-enabled / based)	VR (screen based)
exergame; game based; interactive; system	VR (screen based)
exergame; gamification	MR
game; game based; interactive; program	MR
e-health / m-health	touch-based interaction
Information and Communication Technology / Platform; exergame based; system; technology(-enabled / based)	MR
exergame	VR (screen based)
application; e-health / m-health	touch-based interaction
game	VR (screen based)
exergame	VR (fully immersive)
Information and Communication Technology / Platform; interactive; system	VR (screen based)
experience	MR
application	touch-based interaction
none; program	touch-based interaction
application; system	-
game	-

Evaluation_HMS_Category
No Entry
Cognition (quan); Fitness / Physiological Parameters (quan); Gait / Mobility: dynamic postural control (quan); Stand: static postural control (quan)
Cognition (quan); Dual Task: motor-cognitive (quan); Fitness / Physiological Parameters (quan); Gait / Mobility: dynamic postural control (quan); Questionnaires on Self-Assessment, Health, QoL & Perception of Exercise (quan); Stand: static postural control (quan)
Fitness / Physiological Parameters (quan); Gait / Mobility: dynamic postural control (quan); Stand: static postural control (quan)
Cognition (quan); Dual Task: motor-cognitive (quan); Gait / Mobility: dynamic postural control (quan); Questionnaires on Self-Assessment, Health, QoL & Perception of Exercise (quan); Stand: static postural control (quan)
Balance & Mobility Test Batteries (quan); Cognition (quan); Dual Task: motor-cognitive (quan); Fitness / Physiological Parameters (quan); Gait / Mobility: dynamic postural control (quan); Questionnaires on Self-Assessment, Health, QoL & Perception of Exercise (quan); Stand: static postural control (quan)
No Entry
No Entry
Fitness / Physiological Parameters (quan); Gait / Mobility: dynamic postural control (quan); Stand: static postural control (quan)
Fitness / Physiological Parameters (quan); Gait / Mobility: dynamic postural control (quan); Stand: static postural control (quan)
Balance & Mobility Test Batteries (quan); Fitness / Physiological Parameters (quan); Interview / Questions / Workshops (qual)
Questionnaires on Self-Assessment, Health, QoL & Perception of Exercise (quan)
Balance & Mobility Test Batteries (quan); Cognition (quan); Dual Task: motor-cognitive (quan); Fitness / Physiological Parameters (quan); Gait / Mobility: dynamic postural control (quan); Questionnaires on Self-Assessment, Health, QoL & Perception of Exercise (quan); Self-report falls and exercises (qual); Stand: static postural control (quan)
No Entry
No Entry
Cognition (quan); Dual Task: motor-cognitive (quan); Questionnaires on Self-Assessment, Health, QoL & Perception of Exercise (quan); Stand: static postural control (quan)
Stand: static postural control (quan)
Balance & Mobility Test Batteries (quan); Cognition (quan); Dual Task: motor-cognitive (quan); Fitness / Physiological Parameters (quan); Gait / Mobility: dynamic postural control (quan); Questionnaires on Self-Assessment, Health, QoL & Perception of Exercise (quan); Stand: static postural control (quan)
Gait / Mobility: dynamic postural control (quan)
No Entry
No Entry
Gait / Mobility: dynamic postural control (quan); Interview / Questions / Workshops (qual); Self-report falls and exercises (qual)
Balance & Mobility Test Batteries (quan); Cognition (quan); Dual Task: motor-cognitive (quan); Fitness / Physiological Parameters (quan); Gait / Mobility: dynamic postural control (quan); Questionnaires on Self-Assessment, Health, QoL & Perception of Exercise (quan); Stand: static postural control (quan)
Balance & Mobility Test Batteries (quan); Cognition (quan); Dual Task: motor-cognitive (quan); Questionnaires on Self-Assessment, Health, QoL & Perception of Exercise (quan)
Questionnaires on Self-Assessment, Health, QoL & Perception of Exercise (quan); Stand: static postural control (quan)
Interview / Questions / Workshops (qual); Questionnaires on Self-Assessment, Health, QoL & Perception of Exercise (quan)
No Entry
Interview / Questions / Workshops (qual)
Questionnaires on Self-Assessment, Health, QoL & Perception of Exercise (quan); Self-report falls and exercises (qual)
No Entry
Fitness / Physiological Parameters (quan); Gait / Mobility: dynamic postural control (quan); Stand: static postural control (quan)
No Entry
No Entry
No Entry
No Entry
No Entry
Fitness / Physiological Parameters (quan); Stand: static postural control (quan)
Fitness / Physiological Parameters (quan); Stand: static postural control (quan)
Cognition (quan)
Cognition (quan); Gait / Mobility: dynamic postural control (quan); Questionnaires on Self-Assessment, Health, QoL & Perception of Exercise (quan); Stand: static postural control (quan)
Dual Task: motor-cognitive (quan); Stand: static postural control (quan)
No Entry
Questionnaires on Self-Assessment, Health, QoL & Perception of Exercise (quan); Stand: static postural control (quan)
Balance & Mobility Test Batteries (quan)
Gait / Mobility: dynamic postural control (quan); Stand: static postural control (quan)
No Entry
Balance & Mobility Test Batteries (quan); Fitness / Physiological Parameters (quan); Gait / Mobility: dynamic postural control (quan); Questionnaires on Self-Assessment, Health, QoL & Perception of Exercise (quan); Stand: static postural control (quan)
No Entry
No Entry
Fitness / Physiological Parameters (quan); Questionnaires on Self-Assessment, Health, QoL & Perception of Exercise (quan)
No Entry
No Entry
Expert Assessment (qual)
Fitness / Physiological Parameters (quan); Gait / Mobility: dynamic postural control (quan); Stand: static postural control (quan)
No Entry
No Entry
No Entry
No Entry
No Entry

HMS_top	Evaluation_UXG_Category	UXG_top
No entry	Interviews on Usability & Experience (qual); Questionnaires on Usability (quan); Questionnaires on User / Player Experience (quan)	Evaluation methods
Evaluation methods	No Entry	No entry
Evaluation methods	In Game/System Measurement (quan)	Evaluation methods
Evaluation methods	No Entry	No entry
Evaluation methods	No Entry	No entry
Evaluation methods	Questionnaires on Usability (quan); Technology acceptance (qual + quan)	Evaluation methods
No entry	Interviews on Usability & Experience (qual); Observations & Think Aloud (qual); Questionnaires on Usability (quan); Technology acceptance (qual + quan)	Evaluation methods
No entry	No Entry	No entry
Evaluation methods	No Entry	No entry
Evaluation methods	No Entry	No entry
Evaluation methods	Questionnaires on Usability (quan); Questionnaires on User / Player Experience (quan)	Evaluation methods
Evaluation methods	Interviews on Usability & Experience (qual)	Evaluation methods
Evaluation methods	Questionnaires on Usability (quan)	Evaluation methods
No entry	Interviews on Usability & Experience (qual)	Evaluation methods
No entry	Interviews on Usability & Experience (qual)	Evaluation methods
Evaluation methods	No Entry	No entry
Evaluation methods	In Game/System Measurement (quan); Interviews on Usability & Experience (qual)	Evaluation methods
Evaluation methods	Questionnaires on User / Player Experience (quan)	Evaluation methods
Evaluation methods	Interviews on Usability & Experience (qual); Questionnaires on Usability (quan); Questionnaires on User / Player Experience (quan)	Evaluation methods
No entry	No Entry	No entry
No entry	Interviews on Usability & Experience (qual); Observations & Think Aloud (qual)	Evaluation methods
Evaluation methods	No Entry	No entry
Evaluation methods	Interviews on Usability & Experience (qual); Observations & Think Aloud (qual); Questionnaires on Usability (quan); Questionnaires on User / Player Experience (quan)	Evaluation methods
Evaluation methods	Interviews on Usability & Experience (qual); Observations & Think Aloud (qual); Questionnaires on Usability (quan); Questionnaires on User / Player Experience (quan)	Evaluation methods
Evaluation methods	No Entry	No entry
Evaluation methods	Questionnaires on User / Player Experience (quan)	Evaluation methods
No entry	Interviews on Usability & Experience (qual); Observations & Think Aloud (qual)	Evaluation methods
Evaluation methods	Interviews on Usability & Experience (qual); Observations & Think Aloud (qual)	Evaluation methods
Evaluation methods	Observations & Think Aloud (qual); Questionnaires on Usability (quan); Questionnaires on User / Player Experience (quan); Technology acceptance (qual + quan)	Evaluation methods
No entry	Interviews on Usability & Experience (qual); Questionnaires on Usability (quan); Questionnaires on User / Player Experience (quan)	Evaluation methods
Evaluation methods	No Entry	No entry
No entry	No Entry	No entry
No entry	Interviews on Usability & Experience (qual); Observations & Think Aloud (qual)	Evaluation methods
No entry	Interviews on Usability & Experience (qual); Questionnaires on Usability (quan)	Evaluation methods
No entry	Interviews on Usability & Experience (qual); Observations & Think Aloud (qual); Questionnaires on Usability (quan)	Evaluation methods
No entry	No Entry	No entry
Evaluation methods	In Game/System Measurement (quan); Interviews on Usability & Experience (qual); Questionnaires on User / Player Experience (quan)	Evaluation methods
Evaluation methods	Questionnaires on User / Player Experience (quan)	Evaluation methods
Evaluation methods	No Entry	No entry
Evaluation methods	Questionnaires on Usability (quan); Questionnaires on User / Player Experience (quan)	Evaluation methods
Evaluation methods	No Entry	No entry
No entry	Expert Assessment (qual); Interviews on Usability & Experience (qual); Questionnaires on Usability (quan); Questionnaires on User / Player Experience (quan)	Evaluation methods
Evaluation methods	Questionnaires on User / Player Experience (quan)	Evaluation methods
Evaluation methods	Interviews on Usability & Experience (qual); Observations & Think Aloud (qual)	Evaluation methods
Evaluation methods	Questionnaires on User / Player Experience (quan)	Evaluation methods
No entry	No Entry	No entry
Evaluation methods	No Entry	No entry
No entry	No Entry	No entry
No entry	Interviews on Usability & Experience (qual); Questionnaires on User / Player Experience (quan)	Evaluation methods
Evaluation methods	Questionnaires on User / Player Experience (quan)	Evaluation methods
No entry	Interviews on Usability & Experience (qual); Observations & Think Aloud (qual); Questionnaires on Usability (quan); Questionnaires on User / Player Experience (quan)	Evaluation methods
No entry	No Entry	No entry
Evaluation methods	Interviews on Usability & Experience (qual); Questionnaires on User / Player Experience (quan)	Evaluation methods
Evaluation methods	No Entry	No entry
No entry	No Entry	No entry
No entry	No Entry	No entry
No entry	Interviews on Usability & Experience (qual)	Evaluation methods
No entry	No Entry	No entry
No entry	Interviews on Usability & Experience (qual)	Evaluation methods

Evaluation_type	Evaluation_shared_Category	Evaluation_shared_top
quan + qual	Enjoyment & Motivation for physical activity scales; Flow questionnaires; In game/system measurements: compliance & performance	Evaluation methods
quan	In game/system measurements: compliance & performance	Evaluation methods
quan	No Entry	No entry
quan	In game/system measurements: compliance & performance	Evaluation methods
quan	Enjoyment & Motivation for physical activity scales; In game/system measurements: compliance & performance; Self-reports, interview, supervisor: compliance & performance	Evaluation methods
quan	Enjoyment & Motivation for physical activity scales; In game/system measurements: compliance & performance	Evaluation methods
quan + qual	Enjoyment & Motivation for physical activity scales	Evaluation methods
No entry	No Entry	No entry
quan	No Entry	No entry
quan	No Entry	No entry
mixed methods	No Entry	No entry
quan + qual	No Entry	No entry
quan	Enjoyment & Motivation for physical activity scales	Evaluation methods
qual	No Entry	No entry
qual	Self-reports, interview, supervisor: compliance & performance	Evaluation methods
quan	In game/system measurements: compliance & performance; Self-reports, interview, supervisor: compliance & performance	Evaluation methods
quan + qual	No Entry	No entry
quan	In game/system measurements: compliance & performance; Self-reports, interview, supervisor: compliance & performance	Evaluation methods
quan + qual	Self-reports, interview, supervisor: compliance & performance	Evaluation methods
workshop/focus group	No Entry	No entry
qual	No Entry	No entry
quan + qual	No Entry	No entry
mixed methods	In game/system measurements: compliance & performance	Evaluation methods
mixed methods	Self-reports, interview, supervisor: compliance & performance	Evaluation methods
quan	No Entry	No entry
quan	No Entry	No entry
qual	No Entry	No entry
quan + qual	Enjoyment & Motivation for physical activity scales	Evaluation methods
quan + qual	No Entry	No entry
quan + qual	No Entry	No entry
quan	No Entry	No entry
No entry	No Entry	No entry
qual	No Entry	No entry
quan + qual	Flow questionnaires	Evaluation methods
quan + qual	No Entry	No entry
design process	No Entry	No entry
quan + qual	No Entry	No entry
quan	No Entry	No entry
quan	No Entry	No entry
quan	No Entry	No entry
quan	No Entry	No entry
quan + qual	No Entry	No entry
quan	In game/system measurements: compliance & performance	Evaluation methods
qual	No Entry	No entry
quan	No Entry	No entry
design process	No Entry	No entry
quan	No Entry	No entry
workshop/focus group	No Entry	No entry
mixed methods	No Entry	No entry
quan + qual	Enjoyment & Motivation for physical activity scales	Evaluation methods
quan + qual; workshop/focus group	No Entry	No entry
No entry	No Entry	No entry
quan + qual; workshop/focus group	No Entry	No entry
quan	In game/system measurements: compliance & performance	Evaluation methods
No entry	No Entry	No entry
workshop/focus group	No Entry	No entry
qual	No Entry	No entry
workshop/focus group	No Entry	No entry
qual; workshop/focus group	No Entry	No entry
