# Supplementary material for: Digital interactive experience- and game-based fall interventions for community-dwelling healthy older adults: a cross-disciplinary systematic review
Source: Front Public Health. 2025 Jan 23;12:1489258. doi: 10.3389/fpubh.2024.1489258 (PMC11799000; doi:10.3389/fpubh.2024.1489258)
Supplement: Data Sheet 2 — Search Matrices [file Data_Sheet_2.pdf]

WebofScience

Category	Subcategory	HMS	UXG
Population	HMS Population	elderly, older adults, senior*, age*	elderly, older adults, senior*, age*
	UXG Population	elderly, older adults, senior*, age*	elderly, older adults, senior*, age*
Design Approach	HMS Design	fall*	fall*
	UXG Design	design*, game*, gami*	experience*, centered, interaction, exergam*, user experience, design*, game*, gami*
Intervention	HMS Fall prevention	intervention, treatment, therapy, activit*, prevention, exercise*, task*	training, prevention, exercise*, task*
	UXG Technology	computer, tool, virtual realit*, app*, vr, xr, extended realit*, technolog*	mixed realit*, augmented realit*, interactive, ar, mr, system, kinect*, wii, virtual realit*, app*, vr, xr, extended realit*, technolog*
Outcomes	HMS outcomes	balance, instability, physical, motor*, cogniti*	movement, motor*, cogniti*
	UXG outcomes	fun, quality of life, fear of falling, risk of falling, psychologic*, emotion*, well-being, wellbeing, motivat*	meaning*, usability, engage*, enjoy*, emotion*, well-being, wellbeing, motivat*

ScienceDirect

Category	Subcategory	HMS	UXG
Population	HMS Population	Elderly, Older Adults, Senior	Elderly, Older Adults, Senior
	UXG Population	Elderly, Older Adults, Senior	Elderly, Older Adults, Senior
Design Approach	HMS Design	Fall	Fall
	UXG Design	Game, Gamification	Experience, Exergame, Game, Gamification

Scopus

Category	Subcategory	HMS	UXG
Population	HMS Population	elderly, older adults, senior*, age, aged	elderly, older adults, senior*, age, aged
	UXG Population	elderly, older adults, senior*, age, aged	elderly, older adults, senior*, age, aged
Design Approach	HMS Design	fall*	fall*
	UXG Design	game*, gami*	experience, centered design, interaction design, game*, gami*, exergam*, user experience, experiences
Intervention	HMS Fall prevention	intervention, treatment, therapy, activit*, prevention, exercise*, task*	training, prevention, exercise*, task*
	UXG Technology	computer, tool, virtual realit*, app*, vr, xr, extended realit*, technolog*	mixed realit*, kinect*, wii, augmented realit*, interactive, ar , mr, virtual realit*, app*, vr, xr, extended realit*, technolog*
Outcomes	HMS outcomes	balance, instability, physical, motor*, cogniti*	movement, motor*, cogniti*
	UXG outcomes	fun, quality of life, fear of falling, risk of falling, emotion*, well-being, wellbeing, motivat*	meaning*, usability, engage*, enjoy*, emotion*, well-being, wellbeing, motivat*

Pubmed

Category	Subcategory	HMS	UXG
Population	HMS Population	elderly, older adults, senior*, age, aged	elderly, older adults, senior*, age, aged
	UXG Population	elderly, older adults, senior*, age, aged	elderly, older adults, senior*, age, aged
Design Approach	HMS Design	fall, falling, fall-prevention	fall, falling, fall-prevention
	UXG Design	exer-game*, game*, gami*	experience*, centered design, interaction, exergam*, user, experience, game*, gami*
Intervention	HMS Fall prevention	intervention, treatment, therapy, activit*, prevention, exercise*, task*	training, prevention, exercise*, task*
	UXG Technology	computer, tool, virtual realit*, app, vr, xr, extended realit*, technolog*, application	mixed realit*, augmented realit*, interactive, ar, mr, system, kinect*, wii, virtual realit*, app, vr, xr, extended realit*, technolog*, application
Outcomes	HMS outcomes	balance, instability, physical, motor*, cogniti*	movement, motor*, cogniti*
	UXG outcomes	fun, quality of life, fear of falling, risk of falling, psychologic*, emotion*, well-being, wellbeing, motivat*	meaning*, usability, engage*, enjoy*, emotion*, well-being, wellbeing, motivat*

IEEE

Category	Subcategory	HMS	UXG
Population	HMS Population	elderly, older adults, senior, age, seniors	elderly, older adults, senior, age, seniors
	UXG Population	elderly, older adults, senior, age, seniors	elderly, older adults, senior, age, seniors
Design Approach	HMS Design	fall, falling	fall, falling
	UXG Design	design, game*, gami*	experience, centered, interaction, design, game*, gami*, exergam* , user experience
Intervention	HMS Fall prevention	intervention,treatment, prevention, exercise, task, therapy, activity, activities	prevention, training, exercise, task
	UXG Technology	virtual reality, app, vr, xr, extended reality, computer, technology, tool, technologies, application	mixed reality, virtual reality, augmented reality, interactive, app, vr, ar, mr, xr, extended reality, system, technology, kinect, wii, technologies, application
Outcomes	HMS outcomes	balance, instability, motor*, cogniti*, physical	motor*, cogniti*, movement
	UXG outcomes	fun, quality of life, fear of falling, risk of falling, emotion*, well-being, wellbeing, motivation	meaning*, usability, engage*, enjoy*, emotion*, well-being, wellbeing, motivation

ACM

Category	Subcategory	HMS	UXG
Population	HMS Population	elderly, older adults, senior*, older, age*	elderly, older adults, senior*, older, age*
	UXG Population	elderly, older adults, senior*, older, age*	elderly, older adults, senior*, older, age*
Design Approach	HMS Design	fall*	fall*
	UXG Design	experience*, *centered, interaction, exergam*, user experience	experience*, *centered, interaction, design*, game*, gami*, exergam*, user experience
Intervention	HMS Fall prevention	intervention, treatment, *prevention, exercise*, task*	*prevention, training, game*, exergam*, exercise*, task*
	UXG Technology	virtual realit*, app*, vr, xr, extended realit*, computer, technolog*, ict, tool	mixed realit*, virtual realit*, augmented realit*, interactive, app*, vr, ar, mr, xr, extended realit*, system, technolog*, kinect, wii, ict
Outcomes	HMS outcomes	balance, instability, motor*, cogniti*, physical	motor*, cogniti*, embodiment, movement
	UXG outcomes	fun, emotion*, well-being, wellbeing, quality of life, fear of falling, risk of falling, motivat*	emotion*, well-being, wellbeing, meaning*, usability, engage*, enjoy*, motivat*
