# Supplementary material for: Digital interactive experience- and game-based fall interventions for community-dwelling healthy older adults: a cross-disciplinary systematic review
Source: Front Public Health. 2025 Jan 23;12:1489258. doi: 10.3389/fpubh.2024.1489258 (PMC11799000; doi:10.3389/fpubh.2024.1489258)
Supplement: Data Sheet 3 — Search Strings [file Data_Sheet_3.pdf]

Search string used in WebofScience

((TI=("experience*")) OR (TI=("centered")) OR (TI=("interaction")) OR (TI=("design*")) OR (TI=("game*")) OR (TI=("gami*")) OR (TI=("exergam*")) OR (TI=("user experience")) OR (KP=("experience*")) OR (KP=("centered")) OR (KP=("interaction")) OR (KP=("design*")) OR (KP=("game*")) OR (KP=("gami*")) OR (KP=("exergam*")) OR (KP=("user experience")) OR (AB=("experience*")) OR (AB=("centered")) OR (AB=("interaction")) OR (AB=("design*")) OR (AB=("game*")) OR (AB=("gami*")) OR (AB=("exergam*")) OR (AB=("user experience")) OR (SO=("experience*")) OR (SO=("centered")) OR (SO=("interaction")) OR (SO=("design*")) OR (SO=("game*")) OR (SO=("gami*")) OR (SO=("exergam*")) OR (SO=("user experience")) OR (AK=("experience*")) OR (AK=("centered")) OR (AK=("interaction")) OR (AK=("design*")) OR (AK=("game*")) OR (AK=("gami*")) OR (AK=("exergam*")) OR (AK=("user experience"))) AND ((TI=("fun")) OR (TI=("emotion*")) OR (TI=("well-being")) OR (TI=("wellbeing")) OR (TI=("meaning*")) OR (TI=("quality of life")) OR (TI=("fear of falling")) OR (TI=("usability")) OR (TI=("engage*")) OR (TI=("enjoy*")) OR (TI=("motivat*")) OR (TI=("risk of falling")) OR (TI=("psychologic*")) OR (KP=("fun")) OR (KP=("emotion*")) OR (KP=("well-being")) OR (KP=("wellbeing")) OR (KP=("meaning*")) OR (KP=("quality of life")) OR (KP=("fear of falling")) OR (KP=("usability")) OR (KP=("engage*")) OR (KP=("enjoy*")) OR (KP=("motivat*")) OR (KP=("risk of falling")) OR (KP=("psychologic*")) OR (AB=("fun")) OR (AB=("emotion*")) OR (AB=("well-being")) OR (AB=("wellbeing")) OR (AB=("meaning*")) OR (AB=("quality of life")) OR (AB=("fear of falling")) OR (AB=("usability")) OR (AB=("engage*")) OR (AB=("enjoy*")) OR (AB=("motivat*")) OR (AB=("risk of falling")) OR (AB=("psychologic*")) OR (SO=("fun")) OR (SO=("emotion*")) OR (SO=("well-being")) OR (SO=("wellbeing")) OR (SO=("meaning*")) OR (SO=("quality of life")) OR (SO=("fear of falling")) OR (SO=("usability")) OR (SO=("engage*")) OR (SO=("enjoy*")) OR (SO=("motivat*")) OR (SO=("risk of falling")) OR (SO=("psychologic*")) OR (AK=("fun")) OR (AK=("emotion*")) OR (AK=("well-being")) OR (AK=("wellbeing")) OR (AK=("meaning*")) OR (AK=("quality of life")) OR (AK=("fear of falling")) OR (AK=("usability")) OR (AK=("engage*")) OR (AK=("enjoy*")) OR (AK=("motivat*")) OR (AK=("risk of falling")) OR (AK=("psychologic*"))) AND ((TI=("mixed realit*")) OR (TI=("virtual realit*")) OR (TI=("augmented realit*")) OR (TI=("interactive")) OR (TI=("app*")) OR (TI=("VR")) OR (TI=("ar ")) OR (TI=("MR")) OR (TI=("XR")) OR (TI=("extended realit*")) OR (TI=("computer")) OR (TI=("system")) OR (TI=("technolog*")) OR (TI=("tool")) OR (TI=("kinect*")) OR (TI=("wii")) OR (KP=("mixed realit*")) OR (KP=("virtual realit*")) OR (KP=("augmented realit*")) OR (KP=("interactive")) OR (KP=("app*")) OR (KP=("VR")) OR (KP=("ar ")) OR (KP=("MR")) OR (KP=("XR")) OR (KP=("extended realit*")) OR (KP=("computer")) OR (KP=("system")) OR (KP=("technolog*")) OR (KP=("tool")) OR (KP=("kinect*")) OR (KP=("wii")) OR (AB=("mixed realit*")) OR (AB=("virtual realit*")) OR (AB=("augmented realit*")) OR (AB=("interactive")) OR (AB=("app*")) OR (AB=("VR")) OR (AB=("ar ")) OR (AB=("MR")) OR (AB=("XR")) OR (AB=("extended realit*")) OR (AB=("computer")) OR (AB=("system")) OR

(AB=("technolog*")) OR (AB=("tool")) OR (AB=("kinect*")) OR (AB=("wii")) OR (SO=("mixed realit*")) OR (SO=("virtual realit*")) OR (SO=("augmented realit*")) OR (SO=("interactive")) OR (SO=("app*")) OR (SO=("VR")) OR (SO=("ar ")) OR (SO=("MR")) OR (SO=("XR")) OR (SO=("extended realit*")) OR (SO=("computer")) OR (SO=("system")) OR (SO=("technolog*")) OR (SO=("tool")) OR (SO=("kinect*")) OR (SO=("wii")) OR (AK=("mixed realit*")) OR (AK=("virtual realit*")) OR (AK=("augmented realit*")) OR (AK=("interactive")) OR (AK=("app*")) OR (AK=("VR")) OR (AK=("ar ")) OR (AK=("MR")) OR (AK=("XR")) OR (AK=("extended realit*")) OR (AK=("computer")) OR (AK=("system")) OR (AK=("technolog*")) OR (AK=("tool")) OR (AK=("kinect*")) OR (AK=("wii"))) AND ((TI=("fall*")) OR (KP=("fall*")) OR (AB=("fall*")) OR (SO=("fall*")) OR (AK=("fall*"))) AND ((TI=("balance")) OR (TI=("instability")) OR (TI=("motor*")) OR (TI=("cogniti*")) OR (TI=("physical")) OR (TI=("movement")) OR (KP=("balance")) OR (KP=("instability")) OR (KP=("motor*")) OR (KP=("cogniti*")) OR (KP=("physical")) OR (KP=("movement")) OR (AB=("balance")) OR (AB=("instability")) OR (AB=("motor*")) OR (AB=("cogniti*")) OR (AB=("physical")) OR (AB=("movement")) OR (SO=("balance")) OR (SO=("instability")) OR (SO=("motor*")) OR (SO=("cogniti*")) OR (SO=("physical")) OR (SO=("movement")) OR (AK=("balance")) OR (AK=("instability")) OR (AK=("motor*")) OR (AK=("cogniti*")) OR (AK=("physical")) OR (AK=("movement"))) AND ((TI=("intervention")) OR (TI=("treatment")) OR (TI=("prevention")) OR (TI=("training")) OR (TI=("exercise*")) OR (TI=("task*")) OR (TI=("therapy")) OR (TI=("activit*")) OR (KP=("intervention")) OR (KP=("treatment")) OR (KP=("prevention")) OR (KP=("training")) OR (KP=("exercise*")) OR (KP=("task*")) OR (KP=("therapy")) OR (KP=("activit*")) OR (AB=("intervention")) OR (AB=("treatment")) OR (AB=("prevention")) OR (AB=("training")) OR (AB=("exercise*")) OR (AB=("task*")) OR (AB=("therapy")) OR (AB=("activit*")) OR (SO=("intervention")) OR (SO=("treatment")) OR (SO=("prevention")) OR (SO=("training")) OR (SO=("exercise*")) OR (SO=("task*")) OR (SO=("therapy")) OR (SO=("activit*")) OR (AK=("intervention")) OR (AK=("treatment")) OR (AK=("prevention")) OR (AK=("training")) OR (AK=("exercise*")) OR (AK=("task*")) OR (AK=("therapy")) OR (AK=("activit*"))) AND ((TI=("elderly")) OR (TI=("older adults")) OR (TI=("senior*")) OR (TI=("age*")) OR (KP=("elderly")) OR (KP=("older adults")) OR (KP=("senior*")) OR (KP=("age*")) OR (AB=("elderly")) OR (AB=("older adults")) OR (AB=("senior*")) OR (AB=("age*")) OR (SO=("elderly")) OR (SO=("older adults")) OR (SO=("senior*")) OR (SO=("age*")) OR (AK=("elderly")) OR (AK=("older adults")) OR (AK=("senior*")) OR (AK=("age*")))

Search string used in Pubmed

("experience*" OR "centered design" OR "interaction" OR "game*" OR "gami*" OR "exergam*" OR "user experience" OR "exer-game*") AND ("fun" OR "emotion*" OR "well-being" OR "wellbeing" OR "meaning*" OR "quality of life" OR "fear of falling" OR "usability" OR "engage*" OR "enjoy*" OR "motivat*" OR "risk of falling" OR "psychologic*") AND ("mixed realit*" OR "virtual realit*" OR "augmented realit*" OR "interactive" OR "app" OR "VR" OR "ar " OR "MR" OR "XR" OR "extended realit*" OR "computer" OR "system" OR "technolog*" OR "tool" OR "kinect*" OR "wii" OR "application") AND ("fall" OR "falling" OR "fall-prevention") AND ("balance" OR "instability" OR "motor*" OR "cogniti*" OR "physical" OR "movement") AND ("intervention" OR "treatment" OR "prevention" OR "training" OR "exercise*" OR "task*" OR "therapy" OR "activit*") AND ("elderly" OR "older adults" OR "senior*" OR "age" OR "aged")

Search string used in ACM

((Title:(Experience* OR *centered OR Interaction OR design* OR Game* OR Gami* OR Exergam* OR "User Experience")) OR (Abstract:(Experience* OR *centered OR Interaction OR design* OR Game* OR Gami* OR Exergam* OR "User Experience")) OR (Keywords:(Experience* OR *centered OR Interaction OR design* OR Game* OR Gami* OR Exergam* OR "User Experience"))) AND ((Title:(Fun OR Emotion* OR Well-being OR Wellbeing OR Meaning* OR "Quality of Life" OR "Fear of Falling" OR usability OR engage* OR enjoy* OR motivat* OR "risk of falling")) OR (Abstract:(Fun OR Emotion* OR Well-being OR Wellbeing OR Meaning* OR "Quality of Life" OR "Fear of Falling" OR usability OR engage* OR enjoy* OR motivat* OR "risk of falling")) OR (Keywords:(Fun OR Emotion* OR Well-being OR Wellbeing OR Meaning* OR "Quality of Life" OR "Fear of Falling" OR usability OR engage* OR enjoy* OR motivat* OR "risk of falling"))) AND ((Title:("Mixed Realit*" OR "Virtual Realit*" OR "Augmented Realit*" OR Interactive OR App* OR VR OR "AR " OR MR OR XR OR "Extended Realit*" OR computer OR system OR technolog* OR kinect OR wii OR ict OR tool)) OR (Abstract:("Mixed Realit*" OR "Virtual Realit*" OR "Augmented Realit*" OR Interactive OR App* OR VR OR "AR " OR MR OR XR OR "Extended Realit*" OR computer OR system OR technolog* OR kinect OR wii OR ict OR tool)) OR (Keywords:("Mixed Realit*" OR "Virtual Realit*" OR "Augmented Realit*" OR Interactive OR App* OR VR OR "AR " OR MR OR XR OR "Extended Realit*" OR computer OR system OR technolog* OR kinect OR wii OR ict OR tool))) AND ((Title:(Fall*)) OR (Abstract:(Fall*)) OR (Keywords:(Fall*))) AND ((Title:(balance OR instability OR motor* OR cogniti* OR physical OR embodiment OR movement)) OR (Abstract:(balance OR instability OR motor* OR cogniti* OR physical OR embodiment OR movement)) OR (Keywords:(balance OR instability OR motor* OR cogniti* OR physical OR embodiment OR movement))) AND ((Title:(intervention OR treatment OR *prevention OR training OR game* OR Exergam* OR exercise* OR task*)) OR (Abstract:(intervention OR treatment OR *prevention OR training OR game* OR Exergam* OR exercise* OR task*)) OR (Keywords:(intervention OR treatment OR *prevention OR training OR game* OR Exergam* OR exercise* OR task*))) AND ((Title:(Elderly OR "Older Adults" OR Senior* OR "Older " OR Age*)) OR (Abstract:(Elderly OR "Older Adults" OR Senior* OR "Older " OR Age*)) OR (Keywords:(Elderly OR "Older Adults" OR Senior* OR "Older " OR Age*)))

Search string used in Scopus:

TITLE-ABS-KEY("experience" OR "centered design" OR "interaction design" OR "game*" OR "gami*" OR "exergam*" OR "user experience" OR "experiences") AND TITLE-ABS-KEY("fun" OR "emotion*" OR "well-being" OR "wellbeing" OR "meaning*" OR "quality of life" OR "fear of falling" OR "usability" OR "engage*" OR "enjoy*" OR "motivat*" OR "risk of falling") AND TITLE-ABS-KEY("mixed realit*" OR "virtual realit*" OR "augmented realit*" OR "interactive" OR "app*" OR "VR" OR "ar " OR "MR" OR "XR" OR "extended realit*" OR "computer" OR "technolog*" OR "tool" OR "kinect*" OR "wii") AND TITLE-ABS-KEY("fall*") AND TITLE-ABS-KEY("balance" OR "instability" OR "motor*" OR "cogniti*" OR "physical" OR "movement") AND TITLE-ABS-KEY("intervention" OR "treatment" OR "prevention" OR "training" OR "exercise*" OR "task*" OR "therapy" OR "activit*") AND TITLE-ABS-KEY("elderly" OR "older adults" OR "senior*" OR "age" OR "aged")

Search string used in Science Direct:

“Experience” OR “Exergame” OR “Game” OR “Gamification” OR “Fall” OR “Elderly” OR “Older Adults” OR “Senior”

Search string used in IEEE:

("All Metadata":Experience OR "All Metadata":centered OR "All Metadata":Interaction OR "All Metadata":design OR "All Metadata":Game* OR "All Metadata":Gami* OR "All Metadata":Exergam* OR "All Metadata":User Experience) AND ("All Metadata":Fun OR "All Metadata":Emotion* OR "All Metadata":Well-being OR "All Metadata":Wellbeing OR "All Metadata":Meaning* OR "All Metadata":Quality of Life OR "All Metadata":Fear of Falling OR "All Metadata":usability OR "All Metadata":engage* OR "All Metadata":enjoy* OR "All Metadata":motivation OR "All Metadata":risk of falling) AND ("All Metadata":Mixed Reality OR "All Metadata":Virtual Reality OR "All Metadata":Augmented Reality OR "All Metadata":Interactive OR "All Metadata":App OR "All Metadata":VR OR "All Metadata":AR OR "All Metadata":MR OR "All Metadata":XR OR "All Metadata":Extended Reality OR "All Metadata":computer OR "All Metadata":system OR "All Metadata":technology OR "All Metadata":tool OR "All Metadata":kinect OR "All Metadata":Wii OR "All Metadata":technologies OR "All Metadata":application) AND ("All Metadata":Fall OR "All Metadata":falling) AND ("All Metadata":balance OR "All Metadata":instability OR "All Metadata":motor* OR "All Metadata":cogniti* OR "All Metadata":physical OR "All Metadata":movement) AND ("All Metadata":intervention OR "All Metadata":treatment OR "All Metadata":prevention OR "All Metadata":training OR "All Metadata":exercise OR "All Metadata":task OR "All Metadata":therapy OR "All Metadata":activity OR "All Metadata":activities) AND ("All Metadata":Elderly OR "All Metadata":Older Adults OR "All Metadata":Senior OR "All Metadata":Age OR "All Metadata":seniors)
